# Differentially culturable tubercle bacteria as a measure of tuberculosis treatment response

**DOI:** 10.3389/fcimb.2022.1064148

**Published:** 2023-01-12

**Authors:** Julian S. Peters, Amanda McIvor, Andrea O. Papadopoulos, Tshepiso Masangana, Bhavna G. Gordhan, Ziyaad Waja, Kennedy Otwombe, Matebogo Letutu, Mireille Kamariza, Timothy R. Sterling, Carolyn R. Bertozzi, Neil A. Martinson, Bavesh D. Kana

**Affiliations:** ^1^ Department of Science and Innovation/National Research Foundation Centre of Excellence for Biomedical Tuberculosis Research, The National Health Laboratory Service, School of Pathology, Faculty of Health Sciences, University of the Witwatersrand, Johannesburg, South Africa; ^2^ Perinatal HIV Research Unit, University of the Witwatersrand, Johannesburg, South Africa; ^3^ School of Public Health, Faculty of Health Sciences, University of the Witwatersrand, Johannesburg, South Africa; ^4^ Department of Biology, Stanford University, Stanford, CA, United States; ^5^ Vanderbilt University Medical Center, Nashville, TN, United States; ^6^ Department of Chemistry, Stanford University, Stanford, CA, United States; ^7^ Howard Hughes Medical Institute, Stanford University, Stanford, CA, United States; ^8^ Johns Hopkins University Center for TB Research, Baltimore, MD, United States

**Keywords:** tuberculosis, resuscitation, colony forming units, most probable number (MPN), differentially culturable tubercle bacteria (DCTB)

## Abstract

**Introduction:**

Routine efficacy assessments of new tuberculosis (TB) treatments include quantitative solid culture or routine liquid culture, which likely miss quantification of drug tolerant bacteria. To improve these assessments, comparative analyses using additional measures such as quantification of differentially culturable tubercle bacteria (DCTB) are required. Essential for enabling this is a comparative measure of TB treatment responses using routine solid and liquid culture with liquid limiting dilutions (LLDs) that detect DCTB in sputum.

**Methods:**

We recruited treatment-naïve TB patients, with and without HIV-infection, and serially quantified their sputum for DCTB over the course of treatment.

**Results:**

Serial sputum sampling in 73 individuals during their first 14 days of treatment demonstrated that clearance of DCTB was slower compared to routine solid culture. Treatment response appeared to be characterized by four patterns: (1) Classic bi-phasic bacterial clearance; (2) early non-responders with slower clearance; (3) paradoxical worsening with an increase in bacterial count upon treatment initiation; and (4) non-responders with no change in bacterial load. During treatment, LLDs displayed greater bacterial yield when compared with quantitative solid culture. Upon treatment completion, 74% [46/62] of specimens displayed residual DCTB and within this group, two recurrences were diagnosed. Residual DCTB upon treatment completion was associated with a higher proportion of MGIT culture, GeneXpert, and smear positivity at two months post treatment. No recurrences occurred in the group without residual DCTB.

**Discussion:**

These data indicate that DCTB assays detect distinct subpopulations of organisms in sputum that are missed by routine solid and liquid culture, and offer important alternatives for efficacy assessments of new TB treatments. The residual DCTB observed upon treatment completion suggests that TB treatment does not always eliminate all bacterial populations, a finding that should be investigated in larger cohorts.

## Introduction

Tuberculosis (TB) therapy requires a prolonged duration to clear drug-tolerant persister bacteria not easily eliminated during early treatment. The underlying assumption is that the microenvironments encountered during infection of the human host drives these bacterial populations into non-replicative, drug tolerant states (Turapov et al., 2014; Hu et al., 2015; Gold and Nathan, 2017). Given the growing body of evidence suggesting drug tolerance is associated with eventual emergence of resistance (Liu et al., 2020), further study of these tolerant organisms is essential for formulating new therapeutic approaches to combat drug resistant TB. Definitive evidence for the presence of drug-tolerant persister organisms during TB treatment has been difficult to obtain due to the complex nature of sputum specimens, the exact source of sampling *Mycobacterium tuberculosis* from the lung, and inherent difficulties in detecting and quantifying persister bacteria. Multiple studies have identified bacterial populations in sputum that are unable to form colonies on solid culture but able to grow in liquid media supplemented with growth factors (Mukamolova et al., 2010; Dhillon et al., 2013; Kolwijck et al., 2014; Chengalroyen et al., 2016; Turapov et al., 2016; Saito et al., 2017; Mcaulay et al., 2018; Dusthackeer et al., 2019; Gordhan et al., 2021; Mcivor et al., 2021; Mesman et al., 2021; Zainabadi et al., 2021). These organisms are termed differentially culturable tubercle bacteria (DCTB) (Chengalroyen et al., 2016) or differentially detectable tubercle bacteria (DDTB) (Saito et al., 2017). In addition to being detected in a variety of extra-pulmonary specimens, DCTB have been identified from sputum prior to initiation and during TB treatment, and recently in animal models of TB disease (Mcaulay et al., 2018; Rosser et al., 2018; Zainabadi et al., 2021; Evangelopoulos et al., 2022). Detection of DCTB is facilitated by supplementation of sputum with culture filtrate (CF) from axenically grown *M. tuberculosis*, which stimulates bacterial growth. DCTB display drug tolerance when compared to bacteria cultured on solid media and their recovery can be enhanced with lipid rich media (Turapov et al., 2016; Mesman et al., 2021). The generation of DCTB has been linked to intracellular oxidative stress, which damages macromolecules in the bacillus, leading to the inability to recover on solid media (Saito et al., 2021). These observations suggest that a rigorous analysis of DCTB populations over the full course of TB chemotherapy could provide valuable insight for measuring treatment response.

In previous work with South African TB patients, we demonstrated that treatment-naïve sputum harbors a significant proportion of DCTB and enhanced detection thereof improved *M. tuberculosis* identification in sputum smear negative patients (Chengalroyen et al., 2016). The growth stimulatory effect of CF has been ascribed to resuscitation promoting factors (Rpfs), a group of bacterial growth stimulatory enzymes found in CF (Mukamolova et al., 2010). However, the concurrent observation of sputum samples harboring DCTB that grow independently of stimulation with Rpfs suggests that other enzymes/molecules may be involved in the growth stimulatory effect of CF (Chengalroyen et al., 2016; Gordhan et al., 2021).

The ability to detect treatment response of all bacterial populations in sputum is critical to understanding the efficacy of current and novel TB therapeutics. New anti-TB drugs are currently tested using a standardized Colony Forming Unit (CFU) assay on solid media and automated liquid culture, both of which may not detect DCTB populations. Hence, an assay that detects DCTB, which could be used to determine how these bacteria respond to new treatments, would help in identifying the best drug combinations for treatment shortening. To address this, we determined the treatment response of DCTB in comparison with CFUs and routine measures of bacterial load (GeneXpert, Mycobacterial Growth Indicator Tube [MGIT, Becton Dickinson] and smear microscopy) in a well-characterized prospective clinical cohort of drug susceptible TB patients at two South African sites (Soweto and Matlosana).

## Methods

### Recruitment and sputum sampling

Ethics approval for this study was obtained from the Human Research Ethics Committee of the University of the Witwatersrand (clearance number M120256). Study participants were approached if they had a positive sputum GeneXpert result from a primary health care clinic which was routinely tested in the public sector, usually the National Health Laboratory Service laboratories. Additional inclusion criteria for this study were: adults at least 18 years of age, able to produce a sputum sample of ≥ 3 ml, either evidence of HIV infection or a recent hard copy HIV test result, and no prior history of treatment for TB. Patients with rifampin resistance at baseline and/or characteristics suggesting non-adherence to study protocol were excluded (**Supplementary Table 1**). Following informed consent, spot and overnight (collected early in the morning) sputum samples were collected prior to TB treatment initiation and participants were followed-up during the course of treatment. For the treatment responses analyses, results of overnight sputa are reported here, as we anticipated these would provide the best bacterial yield. Where overnight sputum was not available, or the resulting cultures contaminated, the spot sputum was used. Data from spot samples were also used when assessing standard measures of bacterial load (smear, GeneXpert and MGIT). Data from overnight and spot sputa are reported seperately serial sputum samples were collected whilst the patient was taking TB treatment (acceptable visit windows enclosed in parentheses): 3 (2 – 4), 7 (5 – 10), 14 (12 – 19), 35 (30 – 40), 56 (50 – 65) and 180 days (170-190) after treatment initiation (**Supplementary Figure 1**). Cure was defined by the presence of negative MGIT cultures and smears at day 180. Treatment failure was defined as a positive smear or MGIT culture at five- or six-months post treatment initiation. Disease recurrence was defined as the reoccurrence of TB after cure.

### Liquid limiting dilution assays

**Figure 1:**
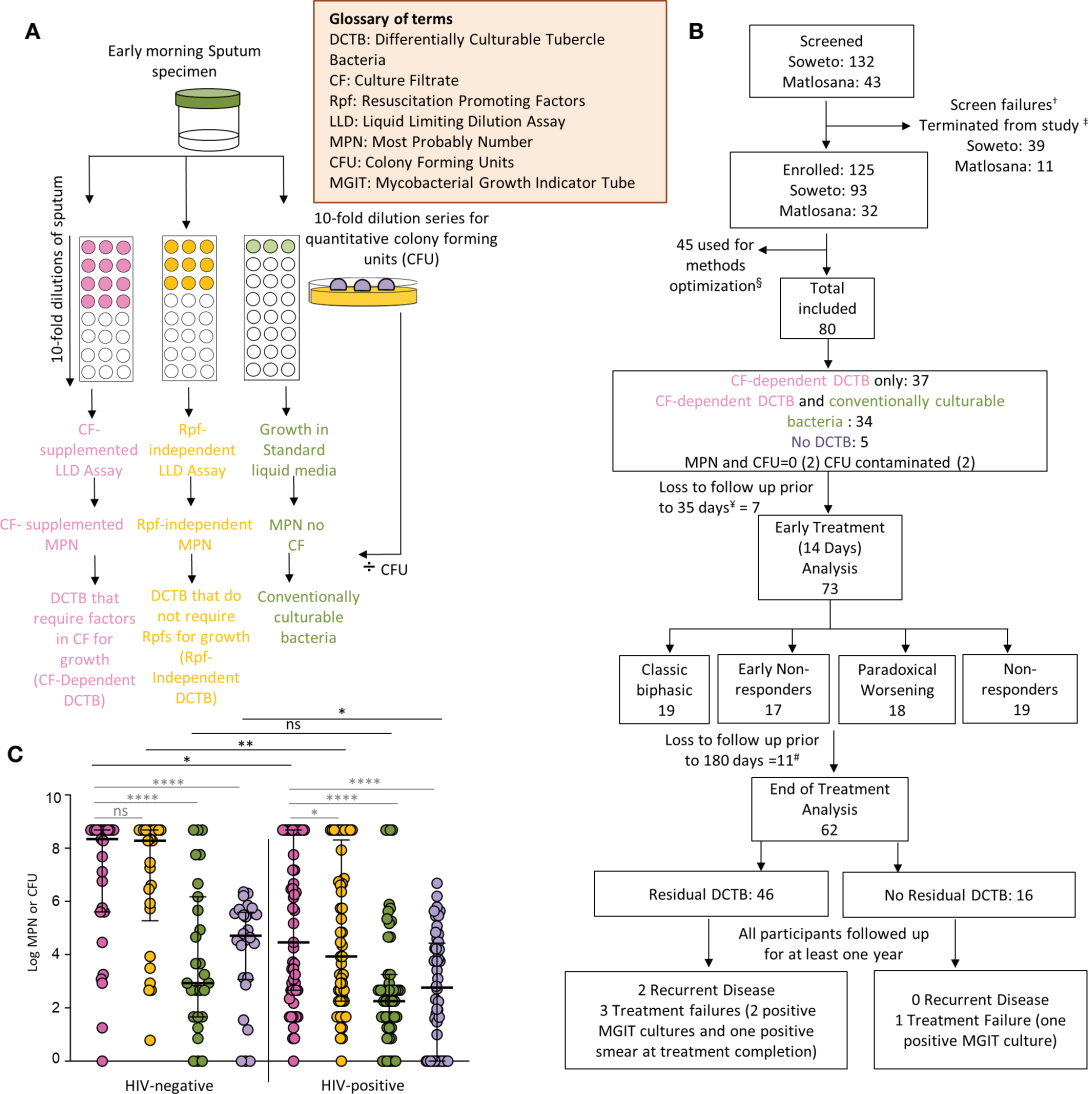
Participant disposition and enrollment microbiology. **(A)** Liquid limiting dilution assays (LLDs) to detect distinct differentially culturable populations in sputum samples. **(B)** Participant disposition flow chart. A total of 175 potential participants were screened for this study from primary health care clinics in the Soweto and Matlosana regions. From each cohort, the arrows depict participants who were removed from the study for various clinical and laboratory reasons. ^†^Screen failures (18 from Soweto, 8 from Matlosana) include potential participants who were initially suitable for the study and then found to not meet the requirements. ^‡^Participants were terminated from the study (21 from Soweto, 3 from Matlosana) and reasons for their removal are documented in the supplementary information. Methods optimization denoted by (^§^) include LLDs performed for sputum samples with different plates and plate-sealing methods to address emerging evaporation of cultures and contamination issues. This aspect is further discussed in the supplementary material. ^¥^Loss to follow up and one participant was excluded as no sputum sample was received for baseline LLD and CFU data. ^#^ One participant missed the day 180 time point but returned thereafter, the individual was excluded from the post 180 day analysis. Data from a total of 80 participants were available for baseline analysis. Additional participants were lost to follow up at various time points throughout the study. Those lost before or after day 35 were classified as early and late dropouts respectively. Data from 73 participants were available for early treatment analysis (sputum samples received up to day 14 following treatment initiation) and data from 62 participants were available for end of treatment analysis (sputum samples received up to 180 days following treatment initiation). **(C)** Bacterial yield using CF-supplemented LLDs (pink), Rpf ^-^ CF-supplemented LLDs (orange), un-supplemented LLDs (green) and colony forming units (purple) in enrollment sputum from TB and TB-HIV infected individuals. *p<0.05, **p<0.01, ****p<0.0001. ns = Not significant.

LLDs entail determination of bacterial count using limiting dilutions of sputum and were carried out as previously described (Chengalroyen et al., 2016). These assays yield the Most probable number (MPN) of bacteria present in a sample. Briefly, CF was obtained from both wild-type and a quintuple *rpf* deletion mutant of *M. tuberculosis* H37Rv strains (Kana et al., 2008) and are referred to as CF and Rpf ¯CF, respectively. Cultures of H37Rv were grown to an Optical Density_600nm_ (OD_600nm_) = 0.6-0.9, from which the cells were harvested by centrifugation at 4 200 x g for 8 minutes and the resulting CF was filtered using a 0.2 µm polyethersulfone (PES) filter (Amicon) to remove any residual cells. To replenish broth nutrients depleted by growing *M. tuberculosis*, CF preparations were diluted 1:1 with fresh Middlebrook 7H9 (Difco) media supplemented with OADC (BD diagnostics) containing 0.05% tween and 450 µl of this was dispensed across 8 wells of a 48 well micro-titre plate (Nunc Thermo) in triplicate. As a control, 450 µl of un-supplemented Middlebrook 7H9 was added across a second 48 well plate in triplicate. To each plate, 50 µl of decontaminated sputum sample was added to the first column of wells and a 10-fold serial dilution was carried out across the plate to the 8^th^ dilution (**Figure 1A**). Growth was assessed at 6 weeks by visual scoring and bacterial counts were calculated using an algorithm based on the Poisson distribution (https://www.wiwiss.fu-berlin.de/fachbereich/vwl/iso/ehemalige/professoren/wilrich/index.html). CFU assays were performed by plating 100 µl of a ten-fold serial dilution series of decontaminated sputum onto Middlebrook 7H11 plates (**Figure 1A**). Further detail can be found in the supplementary material. None of the experimental assays were used to guide participant treatment or care.

### LLD analysis and statistical methods

We assessed the treatment response of bacteria emerging from LLD assays using the MPN value and other readouts. Data were analyzed using Graphpad Prism 7 Medians, interquartile ranges (IQRs) and proportions were determined for continuous measures and categorical variables, respectively. Continuous measures were compared (between CF-supplemented LLDs, Rpf ^-^ CF-supplemented LLDs, un-supplemented LLDs and CFUs) using the Mann-Whitney or Wilcoxon signed-rank tests whereas categorical measures were compared by the Chi-square test. Correlations between pairs of continuous measures were conducted using the Spearman’s rank correlation test. The Kruskal-Wallis test was used to compare the hypothesis of no difference in multiple non-parametric comparisons of LLD derived MPN values and CFU values. LOESS curves were plotted for MPN, CFU, DCTB, the inverse of the MGIT days to positivity (1/MGIT) and the inverse of the GeneXpert cycle threshold (1/GeneXpert). Additional plots were generated and stratified by the following groups: Classic bi-phasic, early non-responders, Paradoxical worsening, and Non-responders. A null hypothesis of no difference in the change in slope over time was tested using linear mixed modelling. The difference between pairs of slopes was determined using an interaction term. SAS Enterprise Guide 7.15 (SAS Institute Inc., Cary, NC, USA) was used to fit the linear mixed models.

## Results

Of 175 individuals screened, 50 (29%) were screen failures (**Figure 1B**, **Supplementary Table 2**). Sputa from 45 (26%) participants were used to optimize LLD culture conditions, which were conducted in real time as sputum was received at the laboratory (**Supplementary Figure 3** and **Supplementary Figure 4**). Therefore, 80/175 (46%) participants in total were included in the baseline analysis, 48 (60%) from Soweto and 32 (40%) from Matlosana. In this cohort, 55/80 (68%) were men, 53/80 (66%) were HIV-positive with a median CD4 T cell count of 167 (IQR 93 – 310) cells/mm^3^ and 21/80 (26%) were +++ smear positive on the World Health Organization grading scale (**Table 1**, which gives statistical results of tests comparing the association between HIV status and demographic, clinical, immunological, microbiological and diagnostic variables). HIV-positive individuals with TB had lower sputum bacterial loads compared to their HIV-negative counterparts as measured by smear status, GeneXpert Cycle threshold, and MGIT culture time to positivity (**Table 1**).

**Table 1:** Demographics, immunology, microbiological and diagnostic data for tuberculosis patients categorized by HIV-1 infection status and C4 T-cell counts.

Variable	Overall (n = 80)	HIV-negative (n = 27)	HIV-positive (n = 53)	p-value
Demographics				
Male, n (%)	55 (68·8)	22 (81.5)	33 (61·8)	0.125*
Female, n (%)	25 (31·3)	5 (18.5)	20 (38·1)	
Age, yr, median (IQR)	36·0 (27·0- 44·0)	27·0 (23 -48·0)	38·5 (31·0 – 43·8)	0.016
**BMI**				
Median at baseline (IQR), kg/m^2^	19·2 (17·9 – 21·6)	18.8 (17·7 – 20.6)	19.4 (18.1 – 22.1)	0.151
**CD4 T-cell count** Median cells/mm3 (IQR)	NA	NA	167·0 (95·5 – 315.5)	
Conventional tuberculosis diagnosis, n (%)				
**Auramine smear**				
Smear grade positive^‡^	45 (56.3)	20 (74.1)	25 (47.1)	0.007^ψ^
Smear grade negative	25 (31.3)	3 (11.1)	22 (41.5)	
Scanty	6 (7.5)	2 (7.4)	4 (7.5)	
Unknown	4 (5)	2 (7.4)	2 (3.8)	
+	13·0 (16.3)	3 (11.1)	10·0 (18·9)	
++	11·0 (13·8)	9 (33.3)	2·0 (3.8)	
+++	21·0 (26.3)	8 (29.6)	13 (24.5)	
GeneXpert result ^§^				
Median GeneXpert cycle threshold (IQR)	19.9 (13.9 – 24.0)	16.5 (13.5·0 – 20·9)	22·3 (14.3 – 26·8)	0.016
High, n (%)	23·0 (28.8)	11·0 (40.7)	12·0 (22·6)	–
Medium, n (%)	16·0 (20)	9·0 (33.3)	7·0 (13.2)	
Low, n (%)	16·0 (20)	3·0 (11.1)	13·0 (24.5)	
Very low, n (%)	8·0 (10)	1·0 (3.7)	7·0 (13.2)	
MTB not detected, n (%)	12·0 (15)	1 (3.7)	11·0 (20.8)	
Unknown	5 (6.3)	2 (7.4)	3 (5.7)	
**Median MGIT time to positivity** days (IQR)	6.5 (4·0 – 14·3)	5·0 (3·0 – 8.0)	9·0 (4·0 – 17·0)	0.018
**Proportion positive MGIT cultures (%)^#^**	63 (90)	22 (96)	41 (87)	

BMI = body mass index; IQR = interquartile range; MGIT = mycobacterial growth indicator tube; NA = not applicable.

* Using the Chi-squared test.

^‡^ Includes scanty, +, ++ and +++.

^ψ^ Using Fishers Exact test (2X2 table).

^§^ GeneXpert: M· tuberculosis was not detected in five patients.

^#^ 70 MGIT culture results were available, 23 for HIV uninfected and 47 for HIV infected.

At enrollment, CF-supplemented LLD assays yielded a statistically significant higher bacterial count when compared to CFUs [log median (IQR) of the CF supplemented LLD = 6.2 (2.9 – 8.7) compared to log median (IQR) CFU = 3.8 (0.0 – 5.1), p<0.0001, (**Figure 1C**)]. LLD assays with standard media yielded a lower bacterial count when compared to CF-supplemented LLDs [LLD without CF: log median (IQR) = 2.7 (1.7 – 4.7), p<0.0001 vs CF+ LLD: log median (IQR) = 6.2 (2.9 – 8.7), **Figure 1C**]. HIV-positive participants had lower CF-supplemented MPN and CFU counts compared to HIV-negative counterparts (**Figure 1C**). In both HIV-positive and HIV-negative individuals, the addition of CF to LLD assays resulted in a statistically significant increase in bacterial yield compared to the CFUs and MPN count without CF-supplementation (**Figure 1C**). CF-dependent DCTB were recovered from 37/80 (46%) baseline specimens, with no bacteria emerging from the same specimens in standard media (**Supplementary Figure 5B**). The quantum of CF-dependent DCTB recovered varied between specimens. In 34/80 (43%) specimens, both CF-dependent DCTB and conventionally culturable organisms were recovered and, in most cases, supplementation of LLD assays with CF yielded higher bacterial counts [CF-supplemented MPN, log median (IQR) = 2.9 (1.9 – 3.4) compared to MPN count with no CF, log median (IQR) = 1.8 (0.8 – 2.7), p=0.0047]. In 5/80 (6.3%) sputum specimens, no DCTB were recovered, with the CFU count yielding higher bacterial loads compared to CF-supplemented and un-supplemented LLD assays (**Supplementary Figure 5B**). In 4/80 (5%) specimens, the MPN count or CFU was negative or the CFU was contaminated. Overall, 71/80 (89%) specimens demonstrated the presence of DCTB at baseline, suggesting a substantive increase in bacterial yield from sputum culture through supplementation of liquid growth assays with CF.

To assess whether the yield of DCTB required the presence of Rpfs in CF, baseline sputum LLDs supplemented with CF were compared with those containing CF from an Rpf-deficient mutant of *M. tuberculosis*. In all specimens, removal of Rpfs from the CF in LLDs yielded a 0.5 log decrease in bacterial count when compared to assays with CF containing Rpfs, suggesting that inclusion of Rpfs in the CF confers a benefit in determining bacterial yield prior to treatment. This effect was statistically significant in HIV-infected individuals only (**Figure 1C**) and given this, we focused on analyzing trends for the LLDs supplemented with CF containing Rpfs in the remainder of this analysis.

**Figure 2:**
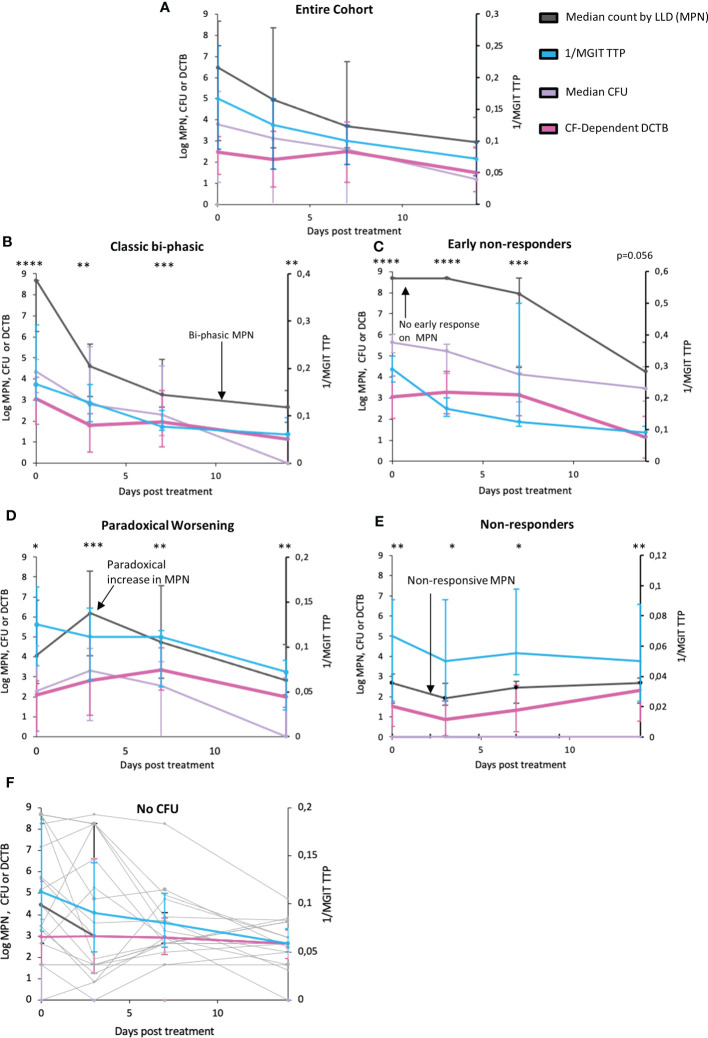
Trends in bacterial clearance over time during standard TB treatment using various measure of bacterial load in sputum. **(A)** Trendlines for bacterial clearance in all sputum specimens. **(B–E)** Bacterial clearance patterns for subcategories including Classic bi-phasic, Early non-responders, Paradoxical worsening, and Non–responsive groups, based on the median trend line of the LLDs. Error bars represent the interquartile range. **(F)** Trends in bacterial clearance in individuals where all no CFUs were recorded across all time points (or there was only 1 time point where there was a readable CFU) within the first two weeks of treatment. Light grey bars represent individual participant MPN values. MPN indicates the most probable number obtained from LLDs assays. *: p<0.05; **: p<0.01; ***p<0.001; ****p<0.0001.

Comparing bacterial clearance from serial sputum sampling over the first 14 days of TB treatment revealed a slower rate of bacterial clearance for DCTB than that reported by CFUs or Mycobacterial Growth Indicator Tube Time to Positivity (MGIT-TTP) (**Figure 2A**, p=0.0735 and p=0.0038 respectively, **Supplementary Table 6** and **Supplementary Figure 6**). Upon visual inspection of treatment response patterns, based on raw MPN counts, four patterns emerged (detailed in **Supplementary Table 5**; individual patient graphs are given in the **Supplementary Figure 11**). The first pattern (n=19), termed “Classic bi-phasic”, was defined by a rapid initial decline in both MPN and CFU counts, during the first week, followed by a slower rate of bacterial clearance thereafter (**Figure 2B**). LOESS curves and linear mixed modelling indicated that DCTB and CFUs cleared at equivalent rates, but there were significant differences in clearance between DCTB and standard liquid culture (**Supplementary Table 6**). The second pattern (n=17), termed “Early non-responders”, was defined by no substantial change (< 0.5 log) in MPN count between the enrolment sample and either at day 3 or at day 7 of treatment, followed by a steady decline up to day 14 of treatment (**Figure 2C**). CFUs and DCTB declined in comparable manner in this group (**Supplementary Table 6**). The third pattern (n=18), termed “Paradoxical worsening”, was characterized by an increase in the bacterial load between enrolment and the 3^rd^ or 7^th^ day of treatment (**Figure 2D**). During the same period, MGIT TTP reported a decline in bacterial counts. The rate of DCTB decline was slower compared to CFU, but this difference was not significant (**Supplementary Table 6**). A fourth group (n=19), termed “non-responders”, was characterized by low initial bacterial counts and yielded non-responsive patterns showing negligible changes in DCTB counts during early treatment (**Figure 2E** and **Supplementary Table 6**). We also conducted a correlation analysis of CF-supplemented MPNs versus CFU counts for all participants, at days 0, 3, 7 and 14. The correlation values are generally low or poor when comparing data collected on the same day (Day 0 vs Day 0, Day 3 vs Day 3 etc) except for CFU and CF-Supplemented MPNs at day 14 (r=-0.34) where the correlation is negative and moderate (**Supplementary Figure 7**). These data indicate that CF-supplemented LLD assays detect populations of bacteria that are distinct from those detected by the CFU assay.

To investigate if clinical or diagnostic characteristics were associated with these four patterns of response, we compared clinical and microbiological measurements between these groups (**Supplementary Table 7**). Non-responder participants had a larger proportion of HIV-positive individuals, were older than the other groups and had more smokers. No differences in BMIs were noted. There were significant differences for both GeneXpert and MGIT TTP at enrolment between the four groups, with the Classic bi-phasic and Early non-responder groups displaying higher bacterial loads prior to treatment initiation. A caveat is that our sample size was small, and that these patterns were visually identified, suggesting that further interpretation of these data requires caution.

In addition, within these four patterns, we also noted participants (n=18) in whom no CFUs were recorded across all time points (or there was only 1 time point where there was a readable CFU) within the first two weeks of treatment. In these individuals, MPN counts and MGIT TTP was able to measure the response to TB therapy (**Figure 2F**, graphs for individual patients in **Supplementary Figure 8**).

**Figure 3:**
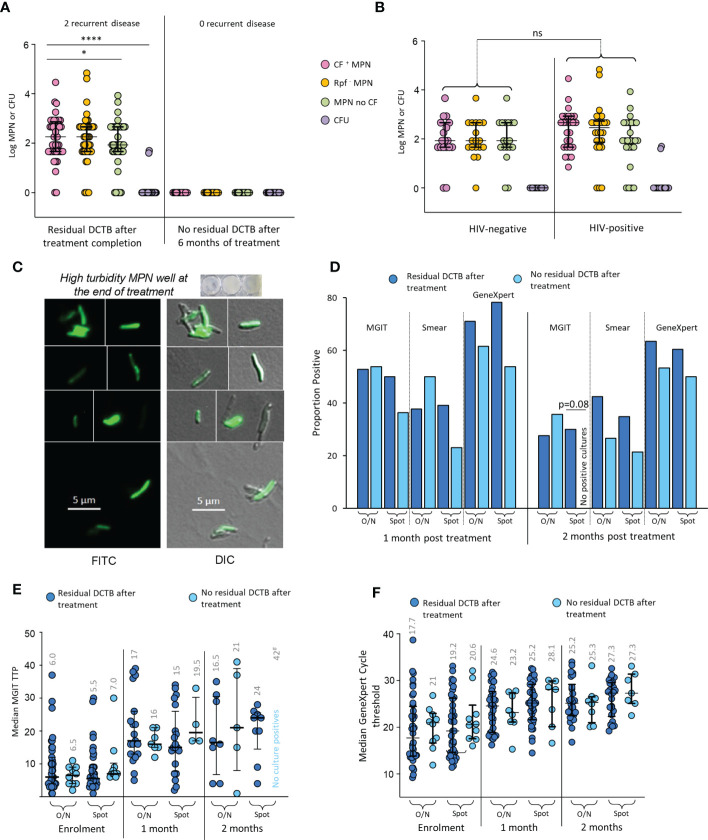
Residual DCTB at the end of treatment. **(A)** Shown is a scattergram of MPN and CFU values in individuals with and without residual DCTB after treatment completion. **(B)** Scattergram of MPN and CFU values for specimens from individuals with residual DCTB after treatment completion stratified by HIV-infection status. Shown are bacterial yields from CF-supplemented LLDs (pink), Rpf ^-^ CF-supplemented LLDs (orange), un-supplemented LLDs (green) and colony forming units (purple). **(C)** Staining of bacteria from end of treatment LLDs using the DMN-Trehalose viability stain, shown is a representative from LLD wells with high turbidity. **(D)** Proportion MGIT, smear and GeneXpert positivity for sputum specimens from individuals with or without residual DCTB after treatment completion. **(E)** Median MGIT time to positivity (TTP) for sputum specimens from individuals with or without residual DCTB after treatment completion. Numbers represent median TTP. **(F)** Median GeneXpert Cycle threshold (CT) for sputum specimens from individuals with or without residual DCTB after treatment completion. Numbers represent median CT. O/N: Overnight specimen, Spot: Spot specimen. * p<0.05; **** p<0.0001. ns = Not significant.

At the end of 6 months of treatment, 41/44 (93.2%) available, non-contaminated MGIT cultures, from early morning sputum were negative for *M. tuberculosis*. The LLD assay (with and without CF) however, identified 46/62 (74.1%) patients whose sputum specimens at treatment completion still contained what appeared to be residual bacteria (**Figure 3A**). In contrast to enrolment data, there were no differences in recovery of DCTB from HIV-infected versus their uninfected counterparts (**Figure 3B**). Within our four categories, the Classic bi-phasic, Paradoxical worsening and early non-responder groups had similar proportions of participants with positive LLD cultures at the end of treatment (6/12 [50%], 11/16 [68.8%], 11/17 [64.7%], respectively). The Non-responder group had a higher proportion of positive LLD cultures (14/17 [82.4%]) upon treatment completion but this difference was not statistically significant. There was no difference in the amount of DCTB recovered between categories (**Supplementary Figure 9**). To assess viability of organisms obtained in the end of treatment LLD assay, we used a fluorescent derivative of trehalose carrying a 4-*N*,*N*-dimethylamino-1,8-napthalimide (DMN) fluorophore, termed DMN-Tre, as a probe for active metabolism (Kamariza et al., 2018). DMN-Tre has a high degree of specificity for mycobacteria and the stain only provides a signal in metabolically active bacilli when incorporated into the mycolic acid layer of viable mycobacterial cells (Kamariza et al., 2018). In 6 randomly selected specimens tested for DMN-Tre staining, rod-shaped organisms were observed in samples derived from CF supplemented LLDs with diverse levels of turbidity, confirming the presence of residual metabolically active *M. tuberculosis* in patient sputum at the end of treatment (**Figure 3C** and **Supplementary Figure 10**).

In individuals with residual DCTB at the end of treatment, a higher proportion had positive MGIT cultures, smears and GeneXpert tests at 2 months after treatment when compared to those without residual DCTB but most of these differences were not statistically significant (**Figure 3D**). We did note a significant difference in MGIT culture positivity with spot specimens where 30% of specimens with DCTB at the end of treatment were positive on MGIT at 2 months versus 0% positivity in specimens with no DCTB. Similarly, median MGIT TTPs at 2 months post treatment were lower (suggesting higher bacterial load) in individuals with residual DCTB after treatment completion (**Figure 3E**). At this time point, there were no differences in GeneXpert cycle threshold, presumably as the DNA of DCTB still gets detected in molecular diagnostics (**Figure 3F**). In the group with residual DCTB after treatment, we recorded 2 recurrent disease episodes within 12 months of treatment completion.

## Discussion

In this prospective cohort of drug susceptible TB patients, we assessed the utility of measuring bacterial load in sputum using LLD assays supplemented with CF as a source of growth stimulatory factors. In most sputum specimens prior to treatment initiation, CF-supplemented LLD assays yielded higher bacterial yield compared to un-supplemented LLD assays and CFUs in both HIV-positive and HIV-negative individuals. In addition to DCTB that required CF to grow, we also found conventionally culturable organisms in sputum as expected. In most cases, these organisms occurred as a mixture of CF-dependent DCTB and conventionally culturable bacteria, underscoring the inherent complexity of bacterial populations in sputum prior to treatment.

Rpfs have been implicated in resuscitation of DCTB in sputum and we observed this in our prior work, however, we also noted the presence of Rpf-independent DCTB (Mukamolova et al., 2010; Chengalroyen et al., 2016). We probed this further in our current study by comparing the yield of DCTB between LLD assays that contained Rpfs to those without. Prior to treatment initiation, a significant requirement for Rpfs to recover DCTB in HIV-infected individuals was noted. These participants had lower bacterial loads than their HIV-uninfected counterparts, suggesting that Rpfs may exert their effects maximally in cases where the bacterial load in sputum is low, an effect that can be exploited to develop better diagnostic tests, particularly for vulnerable groups of individuals. In addition to Rpfs, CF likely contains other molecules that are equally potent in resuscitating DCTB from sputum. Such molecules, including by-products of cell wall cleavage, have been described in literature (Nikitushkin et al., 2012; Shleeva et al., 2014; Nikitushkin et al., 2015). Our prior analysis suggests that the growth stimulatory effect observed with CF is most likely the result of a combination of factors (Gordhan et al., 2021).

During the first 14 days of treatment, CF-supplemented LLDs yielded a significantly greater bacterial count when compared to CFUs. This indicated that in addition to enrolment sputum specimens, sputum from individuals on treatment harbor notable DCTB populations, the detection of which may enable better evaluation of TB treatment response (Zainabadi et al., 2021). This could have important implications for assessing early bactericidal activity (EBA) during the first two weeks of treatment (Diacon and Donald, 2014; Evangelopoulos et al., 2022). EBA studies are the current standard to measure the bactericidal drug activity of new anti-TB drugs or the efficacy of perturbations in current regimens before large phase 3 trials. However, the limitations of EBA to monitor the activity of drugs that have excellent sterilizing capacity but limited bactericidal activity early in treatment are acknowledged (O’brien, 2002; Evangelopoulos et al., 2022). Whilst attempts have been made to improve EBA-type approaches through data modelling, or the use of DNA metrics (Heyckendorf et al., 2017; Gillespie et al., 2002) an alternative assay which describes the ability of drugs to eradicate bacterial persisters has not emerged and remains an urgent priority. MGIT cultures have also been utilized for EBA analyses, however, the four patterns that we have noted in LLD responsive organisms suggests that the standard CFU assay and MGIT culture have limited ability to report on elimination of drug tolerant bacteria such as DCTB. This is further corroborated by the observation that serial sputum specimens from numerous participants in our cohort yielded no CFUs yet had detectable levels of bacteria using LLDs. In cases where patterns of bacterial clearance recorded by LLDs were concordant with those observed by CFUs, the former still yielded a higher bacterial count. In addition, patterns of bacterial decline as measured by LLDs for the Non-responsive and Early non-responder LLD groups differed to those measured by standard MGIT TTP indicating that CF-supplemented LLD assays detect bacterial populations that are missed by standard methods used in the clinical setting. These collective observations could form the basis of development of a new assay to monitor drug activity in individuals who either respond slowly or are unresponsive to therapy.

Using LLDs, residual bacterial growth could be detected in two-thirds of participants at the end of treatment; we confirmed viability of these mycobacteria in a randomly selected subset of specimens using a metabolic probe. As our sample size did not allow for a robust statistical analysis, this observation merits further study. Given that only 2 recurrences were noted in the group of individuals with residual DCTB, it is unlikely that DCTB positivity upon treatment completion is predictive of relapse. Rather, it appears that TB treatment does not eradicate all bacteria and at treatment completion some residual organisms remain. Continued immunological containment of these bacteria would be important to prevent relapse.

Other limitations of our work include the lack of anti-TB drug plasma concentrations to confirm adherence. Whilst directly observed therapy was conducted on days preceding sampling time points, together with an oral report of adherence, other measures of adherence were not done. That said, the majority of patients achieved clinical cure, suggesting that treatment adherence was not a confounder in our study. Furthermore, the use of antiretroviral therapy in HIV-infected participants may have affected bacterial clearance. As we did not collect detailed information on this, no analyses could be done. Also, our separation of treatment response patterns was done using a visual inspection of bacterial clearance patterns. This approach does not exclude the possibility that the groupings arise by random chance, hence we have been cautious in deriving any interpretations from this analysis.

Our study provides an in-depth analysis of DCTB in sputum over the course of TB treatment with findings that may enable the development of new clinical endpoints for measuring drug effectiveness. The superior performance of the DCTB assays in individuals with either low bacterial loads or who respond poorly to treatment underscores the importance of using supplemented liquid cultures for diagnosis of TB, particularly in HIV-endemic settings. This analysis suggests that DCTB assays may be used as a novel measure of treatment response in EBA assays to simultaneously assess both drug tolerant and drug sensitive populations.

## Data availability statement

The original contributions presented in the study are included in the article/**Supplementary Material**, further inquiries can be directed to the corresponding author.

## Ethics statement

The studies involving human participants were reviewed and approved by Human Research Ethics Committee, University of the Witwatersrand. The patients/participants provided their written informed consent to participate in this study.

## Author contributions

BK, JP and AM designed experiments, performed experiments, wrote and edited the manuscript with assistance from NM, CB and TS. AP and TM provided technical assistance, BG managed the study, ZW, ML and NM managed the clinical site operations at the PHRU. KO, NM and TS assisted with statistical analysis. CB and MK aided with metabolic probes and associated data analysis. All authors contributed to the article and approved the submitted version.
