# Supplementary material for: Differentially culturable tubercle bacteria as a measure of tuberculosis treatment response
Source: Front Cell Infect Microbiol. 2023 Jan 12;12:1064148. doi: 10.3389/fcimb.2022.1064148 (PMC9877613; doi:10.3389/fcimb.2022.1064148)
Supplement: Supplementary file 1 [file DataSheet_1.pdf]

Supplementary Material

The response of differentially culturable tubercle bacteria to tuberculosis treatment

Julian S. Peters[1†], Amanda McIvor[1†], Andrea Papadopoulos s[1], Tshepiso Masangana[1], Bhavna G. Gordhan[1], Ziyaad Waja[2], Kennedy Otwombe[2], Matebogo Letutu[2], Mireille Kamariza[3], Timothy R. Sterling[4], Carolyn Bertozzi[5,6], Neil A. Martinson[1,2, 7] and Bavesh D. Kana[1*]

Affiliations:

[1] Department of Science and Technology/National Research Foundation Centre of Excellence for Biomedical TB Research, School of Pathology, Faculty of Health Sciences, University of the Witwatersrand and the National Health Laboratory Service, Johannesburg, South Africa

[2] Perinatal HIV Research Unit, University of the Witwatersrand, Johannesburg, South Africa

[3] Department of Biology, Stanford University, Stanford, CA94305, USA

[4] Vanderbilt University Medical Center, Nashville, TN, Unites States of America (USA)

[5] Department of Chemistry, Stanford University, Stanford, CA94305, USA

[6] Howard Hughes Medical Institute Stanford University, Stanford, CA94305, USA

[7.] Johns Hopkins University Center for TB Research, Baltimore, MD, USA

†These authors contributed equally to this work.

Correspondence: Prof. Bavesh Davandra Kana, DST/NRF Centre of Excellence for Biomedical TB Research, School of Pathology, Faculty of Health Sciences, University of the Witwatersrand and the National Health Laboratory Service, P.O. Box 1038, Johannesburg, 2000, South Africa. Email: bavesh.kana@nhls.ac.za. Telephone: +27 11 489 9030, Fax: +27 11 489 9397

Supplementary Tables

Supplementary Table 1: Patient inclusion and exclusion criteria

Eligibility criteria	Exclusion criteria
• ≥ 18 Years	• < 18 Year
• Able to produce a sputum sample of ≥ 3 ml	• Unable to produce a baseline overnight sample of 3 ml
• A hard copy of an HIV result [a]	• Any form of drug resistance, i.e. Rif mono, MDR-TB, XDR-TB
• Prior lab test result suggestive of TB disease [b]	• Clinical/social characteristics suggestive that the patient would not complete treatment.
• No previous treatment for TB	
• Willing to provide a contact address or phone number	

[a] HIV-negative dated within two months prior to enrolment was accepted. Seropositivity included a two rapid testing algorithm whereby all positive tests were confirmed by a second rapid test on the same specimen, i.e., a formal blood ELISA, a viral load or other molecular assay able to quantify HIV RNA or DNA.

[b] Auramine smear positive (including scanty), culture positive GeneXpert (Rif sensitive only), Hain MTBDR *plus* positive (Rif and INH sensitive only)

Supplementary Table 2. Reasons for removal of patient from analysis (termination)

Reason for termination from study (n = 50 [39 from Soweto, 11 from Matlosana)	Number of patients (%)
**Unspecified reasons**	27
**Relocation**	2
**Consent withdrawal**	6
**History of TB infection**	9
**Death**	1
**Treatment Default**	2
**Lost to follow-up/Missed visits**	3

Supplementary Table 3. Reasons for data used in early treatment analysis, but not available for end-of-treatment analysis

Early/late termination	Reason for no subsequent samples obtained	Number	Time point at which last sputum sample was received (Days)
Early 'drop-outs' used in baseline analysis only (Last sputum sample received before time point 35) n = 7	Hospitalization	2	0, 7
	Missed visit	1	35
	Death	1	14
	Relocation	1	14
	Could not produce sputum	2	7, 7
Late 'drop-outs' Used in baseline and early treatment analysis only (Last sputum sample received at Day 56) n = 10	Relocation	2	35, 56
	Stopped treatment	1	35
	Hospitalized	1	35
	Lost to follow-up	6	35, 56, 56, 56, 56, 56

Supplementary Table 4. PCR primers used to confirm CF preparations from wild type *M. tuberculosis* and an *rpf* gene deletion mutant

Name	Sequence (5'-3')[a]	Application	Amplicon properties/ reference
RpfC-F1	CTCTATCAACGGGCCCTGAC	Forward, reverse and wild type primers used for PCR genotyping of *rpfC* and Δ*rpfC* alleles	371 bp amplicon from wild type *rpfC* and 556 bp amplicon from mutant Δ*rpfC* allele
RpfC-R1	CACAGCAAACCCGAACTCAC		
RpfC-W	GAACTGCAGTCCGCCGTATT		
RpfE-F1	TTATCGTACGGTCCCCTTGG	Forward, reverse and wild type primers for PCR genotyping of *rpfE* and Δ*rpfE* alleles	357 bp amplicon from wild type *rpfE* and 575 bp amplicon from mutant Δ*rpfE* allele
RpfE-R1	TCAGGATCGGCCAGGTCT		
RpfE-W	CGTCGGCATTGGCGATAC		

Supplementary Table 5. Classification criteria used to determine treatment response patterns as measured by LLD.

**Treatment response pattern**	**Criteria used to classify**	**Median (in pink) for CF $^{+}$ MPN as determined by LLDs used to determine treatment response category and corresponding PIDs**
Classic-biphasic	Immediate decline (> 0.5 log) in LLD-derived MPN between treatment initiation and day 3. Decline should sustained, if there is an increase in MPN at any time point, it should not exceed the enrolment MPN	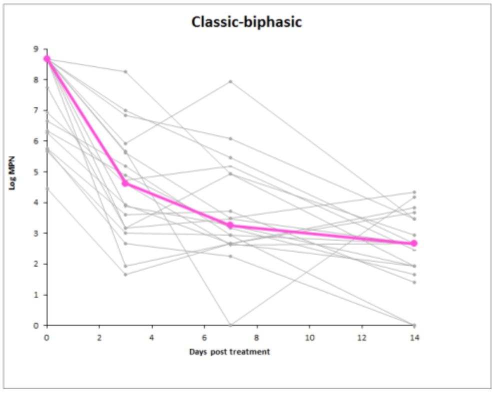 <br>59007, 59012, 59019, 59023*, 59032, 59045, 59047*, 59048, 59125, 59129, 57144, 57145, 57146, 57149, 57159, 57165, 57172, 57173, 57175<br><br>*59047: used in statistical analysis, removed from graph in Supplementary Figure 5 due to bad sample at day 7 (MPN and CFU blank)<br><br>*59023: used in statistical analysis, removed from graph due to an increase in MPN at day 7, however the increase was no more than the enrolment MPN.
Early non-responsive	Less than 0.5 log change in LLD-derived MPN after 3 or 7 days post treatment initiation and a general declining trend after these time points	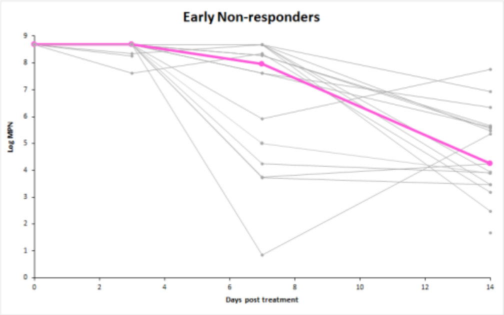 <br>59006, 59010, 59021, 59026, 59031*, 59052, 57163*, 57169, 57174, 59002, 59003, 59004, 59028, 59041, 57158, 57160, 57162*<br><br>*59031: used in statistical analysis, removed from graph due to an increase in MPN at day 14, however

		the increase was no more than the enrolment and day 3 MPN<br><br>*57163: used in statistical analysis, removed from graph Supplementary Figure 5 due to a bad sample received at day 7 therefore an increase in MPN at day 14, however the increase was no more than the enrolment and day 3 MPN<br><br>*57162: used in statistical analysis, due to no sample received at day 7
Paradoxical worsening	An increase in the LLD-derived MPN value above that of the enrolment value, at 3, 7 or 14 days post treatment initiation	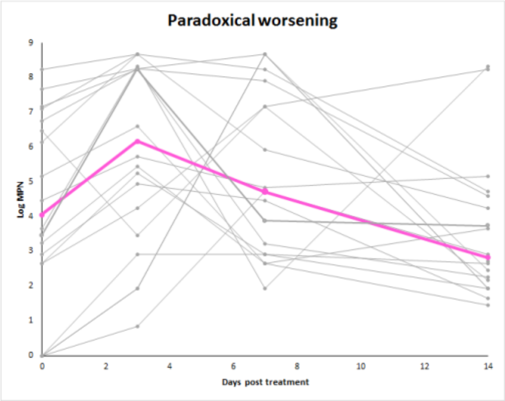<br><br>59005, 59014, 59030, 59035, 59044, 59127, 59128, 57136, 57152, 57154, 57161, 59015, 59016, 59017, 57133, 57134, 57140, 57142
Non-responsive	Negligible (<0.5 log) change in LLD-derived MPN values during the first 35 days of treatment	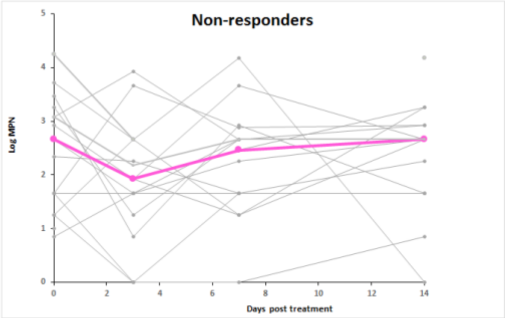<br><br>59018, 59025, 59027, 59029, 59036, 59037, 59038, 59046, 59050, 59051, 59055*, 59056, 57135, 57137, 57139, 57148, 57151, 57155, 57150**59055: used in statistical analysis, removed from graph in Supplementary Figure 5 due to a missed time point at day 7<br><br>*57150: used in statistical analysis, removed from graph due to an unusually high MPN values at

		enrolment day and day 35 that skews the scale of the Y-axis, the overall pattern appeared non-responsive

Supplementary Table 6. LOESS modelling to compare rates of bacterial clearance as measured by different assays

Overall rate of change	LLD	CFU	DCTB	1/MGIT
Combined cohort				
Beta (p-value)	-0.1790 (p<0.0001)	-0.1175 (p<0.0001)	-0.0606 (p=0.0005)	-0.0079 (p<0.0001)
Paradoxical worsening				
Beta (p-value)	-0.09146 (p=0.069)	-0.07963 (p=0.0262)	-0.0077 (p=0.8479)	-0.00177 (p=0.6031)
Early non-responders				
Beta (p-value)	-0.3089 (p<0.0001)	-0.1373 (p<0.0001)	-0.1469 (p<0.0001)	-0.01684 (p=0.0097)
Classic bi-phasic				
Beta (p-value)	-0.3151 (p<0.0001)	-0.2006 (p<0.0001)	-0.1122 (p=0.0015)	-0.01205 (p=0.0003)
Non-responders				
Beta (p-value)	-0.0046 (p=0.8302)	-0.0390 (p=0.1431)	0.03170 (p=0.2405)	-0.0009 (p=0.2967)

**Slope comparison**	**Combined cohort**	**Paradoxical worsening**	**Early non-responders**	**Classic bi-phasic**	**Non-responders**
MPN vs. CFU	P=0.0090	P=0.7686	P<0.0001	P=0.0168	P=0.2425
DCTB vs. CFU	P=0.0735	P=0.3145	P=0.2690	P=0.1043	P=0.0758
1/MGIT vs. CFU	p<0.0001	P=0.0867	P=0.0020	P<0.0001	P=0.1961
DCTB vs. 1/MGIT	p=0.0038	p=0.8776	p<0.0001	p=0.0194	p=0.2433

Supplementary Table 7. Characteristics of participants from different treatment response groups

Variable	Classic biphasic (n=19)	Early non-responders (n=17)	Paradoxical worsening (n=18)	Non-responders (n=19)	p value
**Demographics**					
Male, n (%)	13 (68·4%)	15 (88·2%)	9 (50·0%)	15 (78·9%)	
Female, n (%)	6 (31·6%)	2 (11·8%)	9 (50·0%)	4 (21·1%)	
Age, yr, median (IQR)	35·0 (27·0-45·0)	27·0 (23·5-31·5)	36·5 (27·8-45·0)	43·0 (39·0-52·0)	0.0004[ll]
**BMI**					
Median at baseline (IQR), kg/m^2	18·3 (17·6-19·9)	18·6 (17·3-21·5)	19·9 (18·3-22·2)	20·2 (18·3-23·8)	0.0751
**HIV status, number** (%)	Negative 6 (31·6%)	Negative 9 (52·9%)	Negative 8 (44·4%)	Negative 2 (10·5%)	
	Positive 13 (68·4%)	Positive 8 (47·1%)	Positive 10 (55·5%)	Positive 17 (89·5%)	<0.05*
**Smoking status**	Yes 7 (36·8%)	Yes 6 (35·3%)	Yes 4 (22·2%)	Yes 10 (52·6%)	No difference between groups
**CD4 T-cell count** Median cells/mm3 (IQR)	177 (102.5-341)	171 (95·3-362)	110·5 (53·8-405·3)	168 (97·5-336·5)	No difference between groups
**Conventional tuberculosis diagnosis, n (%)**					
**Auramine smear**					
Smear grade positive [‡]	17 (89.5%)	16 (94.1%)	10 (55.6%)	1 (5.3%)	<0.005[Π]
Smear grade negative	0 (0%)	0 (0)	7 (38.9%)	14 (73.7%)	
Scanty	1 (5.3%)	0 (0)	1 (5.6%)	3 (15.8%)	
Unknown	1(5.3%)	1 (5.9%)	0	1 (5.3%)	
+	4 (21.1%	1 (5.9%)	6 (33.3%)	1 (5.3%)	
++	6 (31.6%)	2 (11.8%)	2 (11.1%)	0 (0)	
+++	7 (36.8%)	14 (82.4%)	2 (11.1%)	0 (0)	
**GeneXpert result** [§]					
Median GeneXpert cycle threshold (IQR)	19.7 (14.8; 21·3)	13·6 (11.0; 14.2)	21.2 (17.7; 26.8)	27.4 (23.0; 29.9)	<0.0001[ll]
High, n (%)	6 (31.6%)	15 (88.2%)	2 (11.1%)	0 (0%)	
Medium, n (%)	8 (42.1%)	1 (15.9%)	7 (38.9%)	0 (0%)	
Low, n (%)	3 (15.8%)	0 (0)	5 (27.8%)	7 (36.8%)	
Very low, n (%)	0 (0)	0 (0)	1 (5.6%)	5 (26.3%)	
MTB not detected, n (%)	0 (0)	0 (0)	3 (16.7%)	6 (31.6%)	
Unknown	2 (10.5%)	1 (5.9%)	0 (0)	1 (5.3%)	
**Median MGIT time to positivity** days (IQR)	5 (3; 7.5)	3.5 (3; 4)	8 (6; 13)	15 (11; 42)	<0.0001[ll]
**MPN**					
Median log CF^+ MPN (IQR)	8·7 (6·3; 8·7)	8·7 (8·7; 8·7)	4·1 (2·7; 6·8)	2·7 (1·7; 3·1)	<0.0001
Median log MPN no CF (IQR)	2·9 (1·7; 5·3)	4·7 (2·5; 8·2)	2·5 (0·0; 3·7)	1·7 (0·9; 2·7)	0.0001
**CFU**					
Median log CFU (IQR)	4·3 (3·8; 5·5)	5·6 (5·1; 6·0)	2·3 (0·0; 4·5)	0·0 (0·0; 2·2)	<0.0001

BMI = body mass index; CF = culture filtrate; IQR = interquartile range; MGIT = mycobacterial growth indicator tube; LLD = Liquid Limiting Dilution Assay. MPN = most probable number; CFU = colony forming unit; NA = not applicable

[ll] Median age, GeneXpert cycle threshold and MGIT TTP were statistically different between groups by ANOVA(Kruskal-Wallis)

[‡] Includes scanty, +, ++ and +++

[Π] p<0.005 between non-responder and all other groups when compare

* p<0.05 between non-responder and early non-responder/paradoxical worsening groups. No statistical difference between non-responder and classic biphasic group. No difference between classic biphasic, early non-responsive and paradoxical worsening groups.

† All the proportion comparisons by HIV status or CD4 T-cell count were conducted using the chi-square test.

§ GeneXpert: *M· tuberculosis* was not detected in five patients (HIV positive subgroup). These patients were omitted from column statistics, i.e. HIV negative n = 25; HIV positive n = 50, overall column statistics for HIV comparison obtained from 75 patients.

Supplementary Methods and Figures

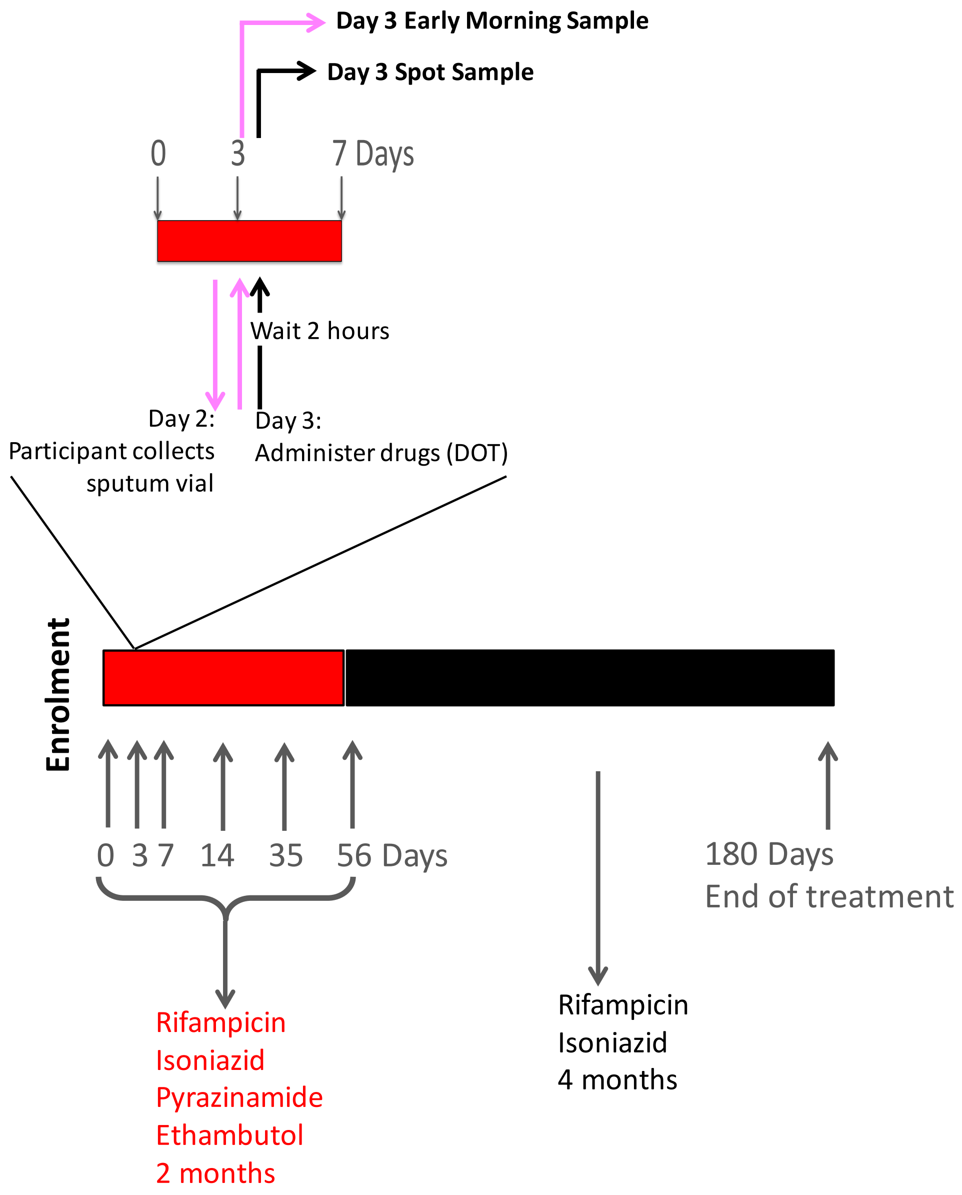

Supplementary Figure 1. Schedule of longitudinal sputum sampling during standard tuberculosis treatment. The inset above the timeline indicates sampling of sputum at day 3 to illustrate sampling approach at all time points. Pink lines indicate the early morning sputum sampling approach and black lines indicate the spot sputum sampling approach.

Processing of sputum samples. Following specimen collection, sputum samples were immediately transferred to the laboratory and decontaminated. Sputum decontamination was performed by the addition of an equal volume of NaOH in NALC-NaOH-sodium citrate solution (or Mycoprep) to the sputum sample (final concentration of NaOH was 1%), followed by centrifugation at 3000 x g for 15 minutes. The pellet was subsequently washed and re-suspended in approximately 4 mL of 0.01M phosphate buffered saline (PBS), pH 7.4. Thereafter, the pellet was split and half of the sample was used for routine diagnostic testing (smear microscopy, GeneXpert and MGIT), while the remainder was used in CFU and LLD assays. The 2 mL of decontaminated sputum earmarked for LLDs and CFU assays was vortexed for approximately ten seconds in the presence of 2 mm glass beads to ensure that bacterial clumps or cords were broken up.

Bacterial culturing procedures. Axenic cultures of wild-type *M. tuberculosis* (H37Rv Johannesburg) (2) and a mutant strain deficient in all five *rpf* genes (BG1 ΔACBED) (3) were prepared by the addition of 1 mL of freezer stock (OD_{600nm} = 0.5 – 0.7) to 8 mL of 7H9 media and grown for 2 days to an OD_{600nm} of approximately 0.5. Fresh 50 mL cultures were prepared daily by the addition of pre-culture (8 mL) to 7H9 media (to 42 mL) supplemented with 0.05% Tween and OADC [oleic acid, albumin, dextrose and catalase, Becton Dickinson, RSA (BD)]. Cultures were grown for 2 to 4 days to an OD_{600nm} = 0.6 to 0.9. The cells from the wild-type and mutant cultures were subsequently harvested by centrifugation (3900 x g, 10 minutes) after which culture filtrate was obtained by filtering the resulting supernatants through a 0.22 µm filter. CF and RPF ¯ CF were obtained from the H37Rv and BG1 strains, respectively. The culture filtrate was then diluted in a 1:1 ratio with 7H9 media and supplemented with 8% PANTA (w/v) (polymixin, amphotericin B, nalidixic acid, trimethoprim and azolocillin, BD). Additionally, a 7H9 media control supplemented with 8% PANTA was prepared (w/v).

Contamination checks. To assess the sterility of the CF and Rpf - CF, a 1 mL aliquot of each preparation was spread onto 7H11 plates and incubated at 37˚ C for up to six weeks. An additional 1 mL aliquot from each culture filtrate was placed into a 1.5 ml Eppendorf tube and incubated for three months to ensure the absence of growth.

PCR confirmation of CF. An additional aliquot of each culture filtrate was frozen for PCR confirmation of the genetic background of the stains and to rule out any possibility of cross contamination. For this, the *rpfC* and *rpfE* genes were amplified using primers given in Table S4. This test simultaneously confirmed the absence of Rpfs in the BG-1 CF. Supplementary Figure 2 depicts the approach and result for *rpfC*. PCR cycling conditions were initial denaturation at 95 ºC for 5 min, followed by 30 cycles of 95 ºC for 30 sec, 60 ºC for 30 sec and 72 ºC for 30 sec, this was followed by a final extension at 72 ºC for 10 min.

CFU assays. For CFU assays, ten-fold serial dilutions were performed by adding 100 µL of decontaminated pellet to 1.5 mL Eppendorf tubes containing 900 µL of 7H9 broth. Serial dilutions were performed for each sample. Thereafter, 100µl of neat and diluted samples were plated out in duplicate on 7H11 agar plates. Before plating, samples were vortexed for approximately ten seconds to ensure adequate dispersion of organisms in the suspension. Plates were placed in breathable bags and placed in the incubator at 37˚ C for up to four weeks.

LLD assays. LLD assays were performed as previously described (4). Briefly, 450 µL of diluted CF-supplemented media (CF from either H37Rv and BG1) was aliquoted in triplicate into a 48-well microtitre plate. Additionally, 450 µl of un-supplemented media was added in triplicate to a second 48-well microtitre plate. Following this, 50µL of decontaminated sputum sample was added to the first well of each assay and diluted 10-fold across the LLD plate. Plates were sealed with plastic tape and placed in the incubator at 37˚C in zip-lock bags. Plates were scored weekly for up to six

weeks after which an aliquot from positive LLD wells was frozen for genotyping. The MPN value of bacterial load, as derived from the LLD assay, was calculated using software (available from

http://www.wiwiss.fu.berlin.de/fachbereich/vwl/iso/ehemalige/wilrich/index.html).

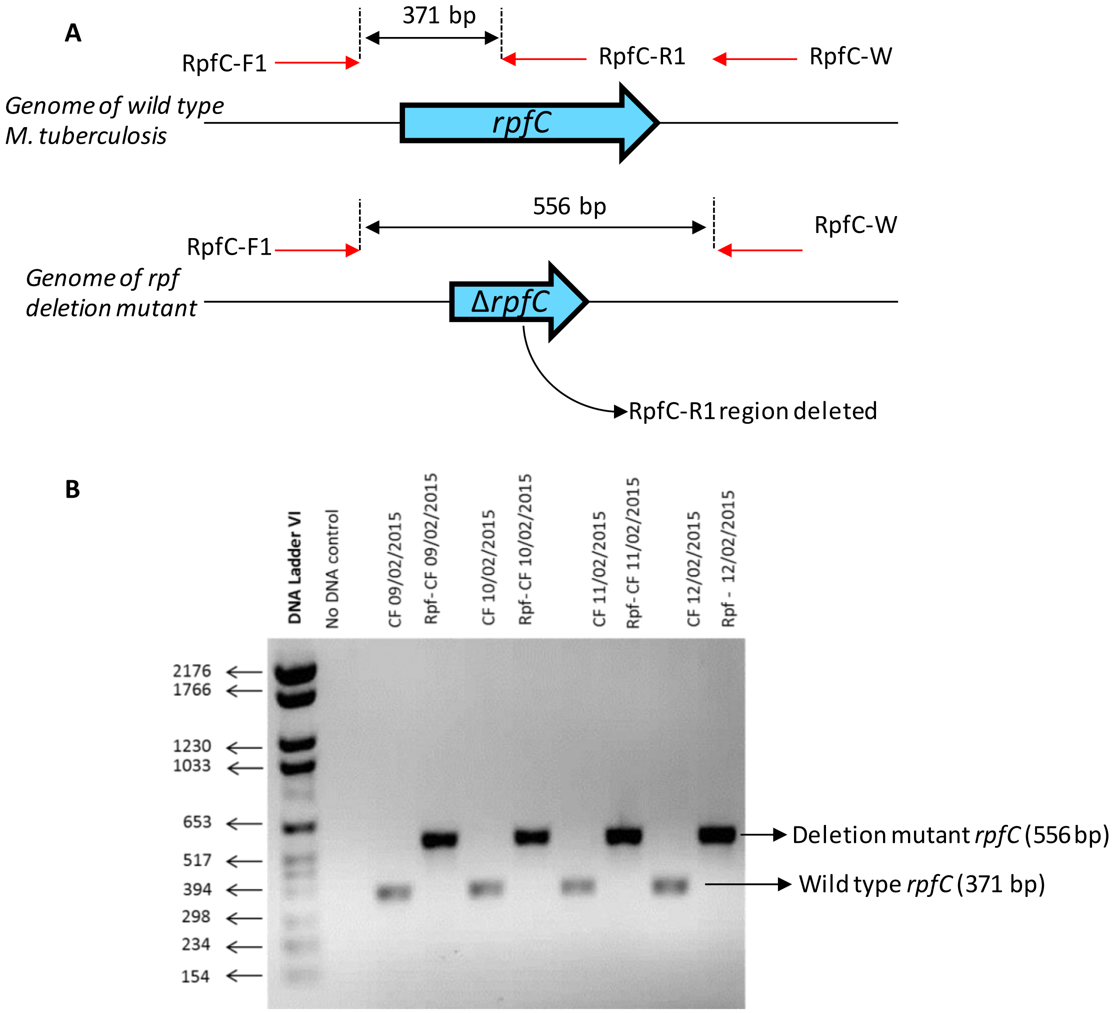

Supplementary Figure 2: PCR confirmation of CF and Rpf- CF preparations. To ensure that CF preparations were not inadvertently mixed, through technician error during LLD assay set up, all preparations of CF from wild type and the *rpf* deletion mutant of *M. tuberculosis* were tested for the presence of either wild type or deletion *rpf* alleles. Two genes, *rpfC* and *rpfE* were probed in this quality assurance test. Shown here is the approach and result for *rpfC*. (A) Depicts the genomic map of the *rpfC* locus in wild type and the *rpf* deletion mutant, with PCR primer binding sites. Primers and their target regions are given in Supplementary Table 4. (B) An example of the PCR result from a test of different preparations of CF. The different lanes represent CF-preparations on different days. Expected sizes are given.

LLD assay refinement. During processing of sputum specimens, we noted condensation on the lid of the 48-well plates used for LLD assays making visual scoring of growth difficult. To address this, we tested the utility of a different brand of 48-well microtitre plate (Biolite, compared with Nunc, which was used previously) that was sealed with autoclave tape after the LLD assay was set up. Routinely, plastic tape was used with Nunc plates. We hypothesized that the autoclave tape would provide a seal that was more prone to gaseous exchange when compared to the plastic tape, thus reducing condensation. The combination of Biolite plates and autoclave tape substantially reduced condensation (Supplementary Figure 3). Considering this, LLD assays using this approach were set up with sputum specimens however, this yielded poor baseline MPN data. Generally, prior to treatment initiation, MPN values exceed CFUs but the combination of Biolite plates with autoclave tape yielded MPN values lower than CFUs. As an example, in baseline sputum very low MPN counts in specimens that had > 4 log CFUs were obtained. At the very least we expect that LLD assays would yield a bacterial count equivalent to the CFU (Supplementary Figure 4A). To further evaluate this, we tested limiting dilutions of an axenic laboratory culture of *M. tuberculosis* H37Rv and found that the combination of Biolite plates, sealed with autoclave tape inhibited bacterial growth when compared to Nunc plates, sealed with plastic tape (Supplementary Figure 4B). Therefore, we reverted to using Nunc plates, sealed with plastic tape for sputum specimens and addressed the condensation issue by gently tapping the lid before scoring. Using this approach robust MPN counts were obtained again in baseline sputum (Supplementary Figure 3A). As a result of this optimization of plate set up, we excluded all patients with specimens that were processed on the Biolite plates sealed with autoclave tape.

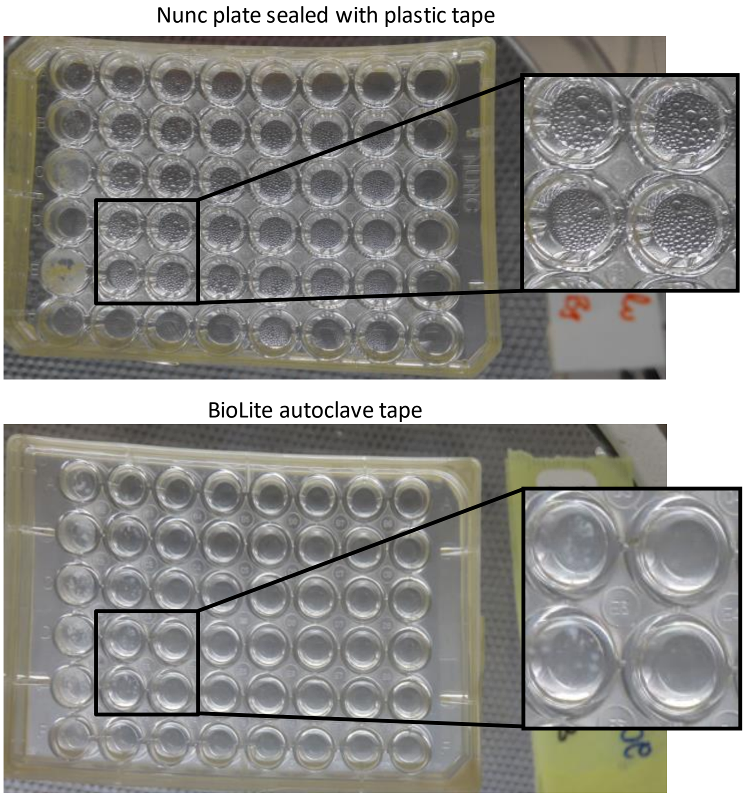

Supplementary Figure 3. The upper panel is a representative depiction of LLD assays in Nunc plates sealed with plastic tape. Condensation on the lid of the plate was common and made scoring of the MPNs difficult. The lower panel represents LLD assays conducted on Biolite plates, sealed with autoclave tape, this combination reduced condensation problems.

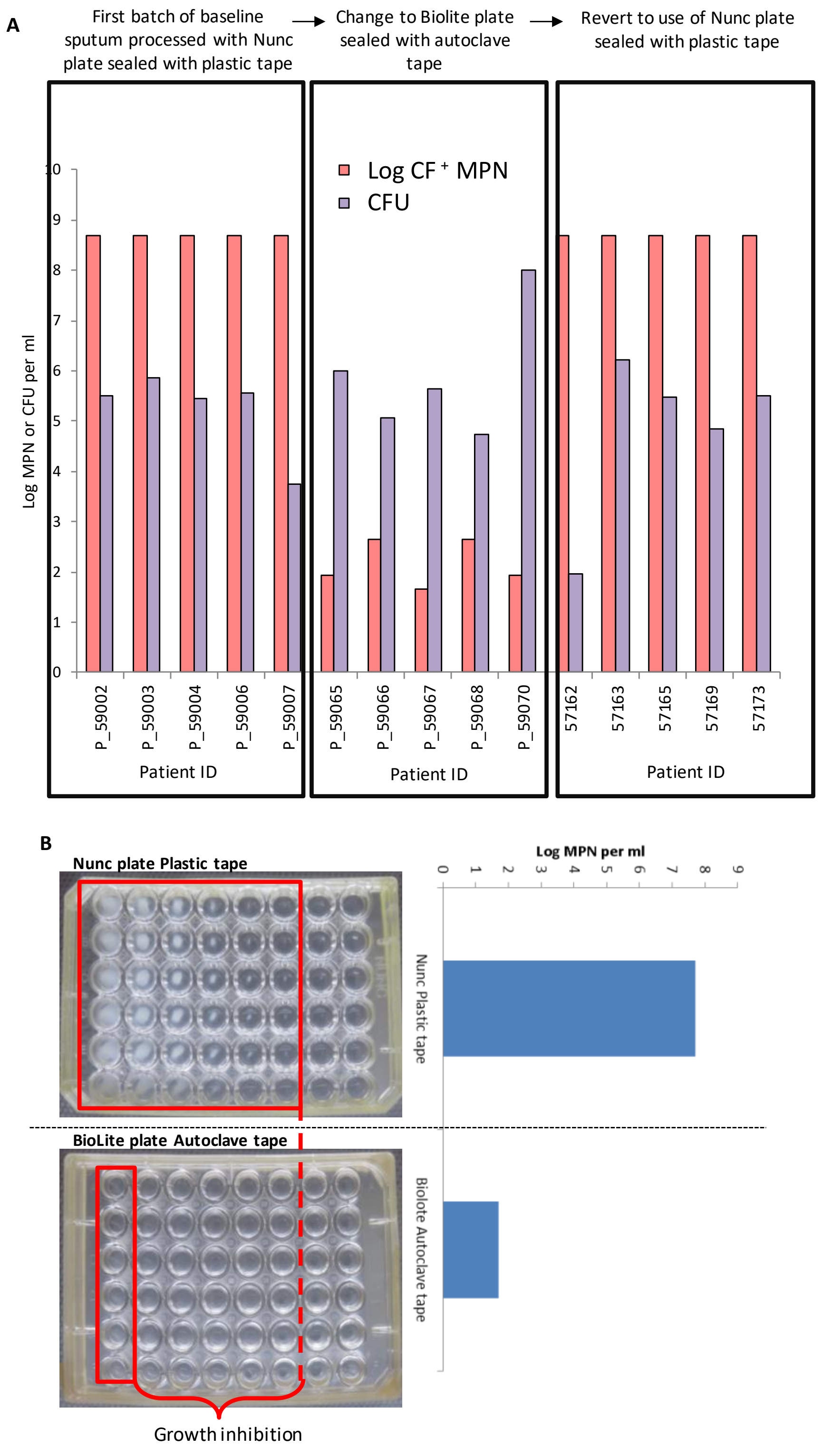

Supplementary Figure 4. Inhibition of bacterial growth in Biolite plates. **(A)** MPN and CFU values for enrolment sputum specimens using different plate formats. **(B)** LLDs using an axenic culture of *M. tuberculosis* in the two plate formats. It has been demonstrated that axenic cultures of *M. tuberculosis* have equivalent levels of CFU and MPN counts and hence, no DCTB (1). When splitting the same culture over the two plates formats, we noted that Biolite plates sealed with autoclave tape inhibited growth of *M. tuberculosis*. Considering this, we reverted to using Nunc plates sealed with plastic tape.

DMN-tre staining and fluorescence microscopy. Staining with a 4-*N*,*N*-dimethylamino-1,8-naphthalimide–conjugated trehalose (DMN-Tre) stain was carried out as previously described (5). Briefly, the dye was added to 100 µl of stored sputum samples cultured in serial dilutions in the 48-well plate LLD format described above. The dye was added to a final concentration of 1 mM and the cells were incubated for 3 hours at 37 ºC to allow for incorporation of the dye into the cells. The cells were fixed with glutaraldehyde, mounted onto an agarose pad on a microscope slide and viewed using the FITC (green) channel using a fluorescent microscope.

Data analysis. Analysis was performed for patients with available MPN, CFU and clinical data (refer to patient disposition flowchart). The bacillary load measures (CF, Rpf- CF, MPN no CF and CFU), MGIT time to positivity (TTP), smear grading and GeneXpert cycle threshold (CT) values were compared between groups and are reported as median and interquartile ranges (Table 1). Patients were further stratified by their HIV status as well as the area from which they were recruited, i.e. Matlosana vs Soweto cohort. Statistics was carried out using GraphPad Prism version 7, Statistica version 12, and R version 3.3.1. Scatterplots depicted the inter-quartile range and the medians. P-values were calculated using means in Mann Whitney's unweighted two-tailed non-parametric tests. Significant differences were considered if the p-value was less than 0.05. Correlation tests were performed using Spearman rank sum correlation and correlation scores were considered significant if the p-value was less than 0.05 at a 0.95 CI.

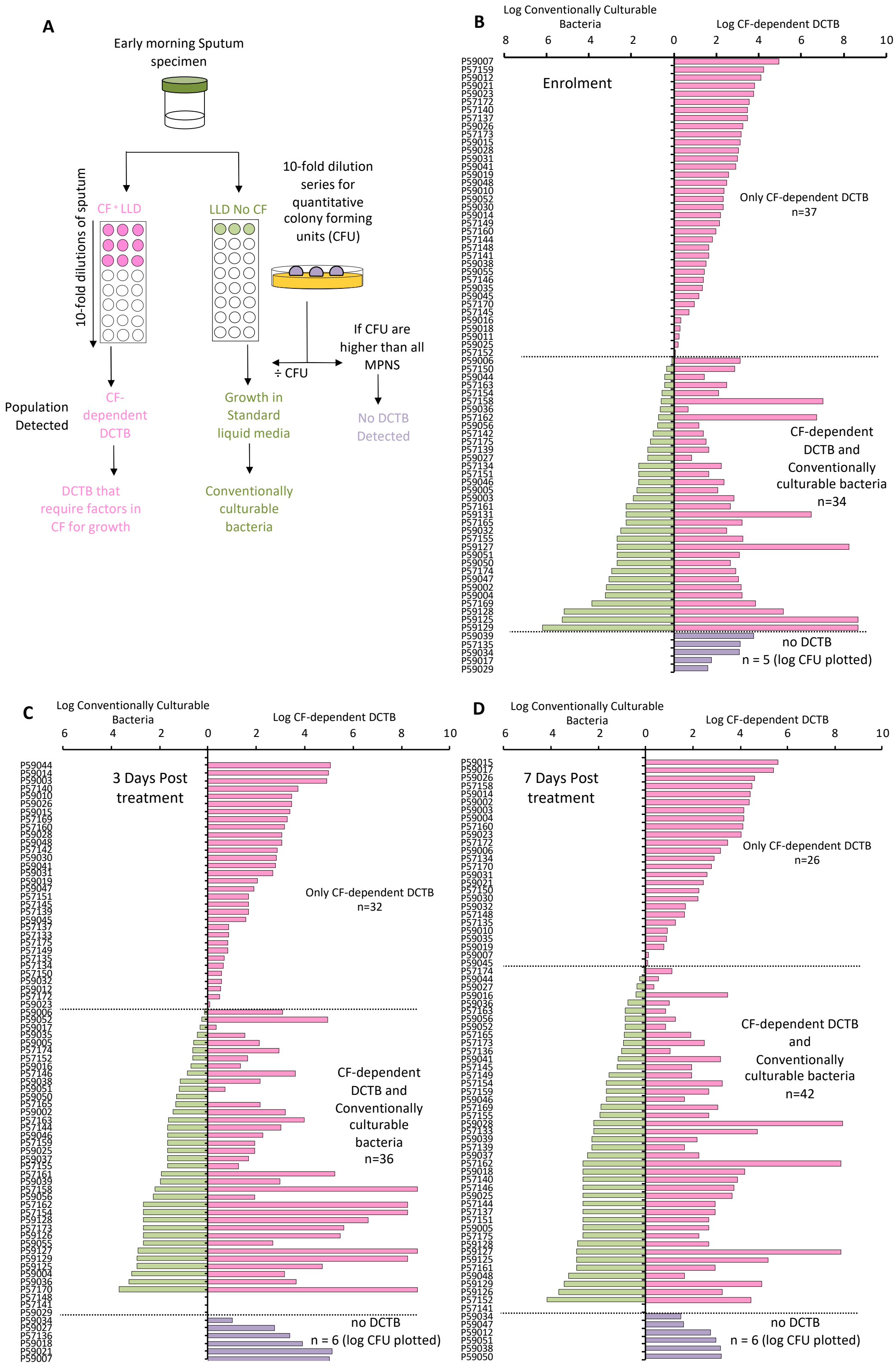
A
Early morning Sputum specimen
10-fold dilutions of sputum
CF⁺ LLD
LLD No CF
10-fold dilution series for quantitative colony forming units (CFU)
If CFU are higher than all MPNS
÷ CFU
Population Detected
CF-dependent DCTB
Growth in Standard liquid media
No DCTB Detected
DCTB that require factors in CF for growth
Conventionally culturable bacteria
B
Log Conventionally Culturable Bacteria
Log CF-dependent DCTB
Enrolment
Only CF-dependent DCTB n=37
CF-dependent DCTB and Conventionally culturable bacteria n=34
no DCTB
n = 5 (log CFU plotted)
C
Log Conventionally Culturable Bacteria
Log CF-dependent DCTB
3 Days Post treatment
Only CF-dependent DCTB n=32
CF-dependent DCTB and Conventionally culturable bacteria n=36
no DCTB
n = 6 (log CFU plotted)
D
Log Conventionally Culturable Bacteria
Log CF-dependent DCTB
7 Days Post treatment
Only CF-dependent DCTB n=26
CF-dependent DCTB and Conventionally culturable bacteria n=42
no DCTB
n = 6 (log CFU plotted)

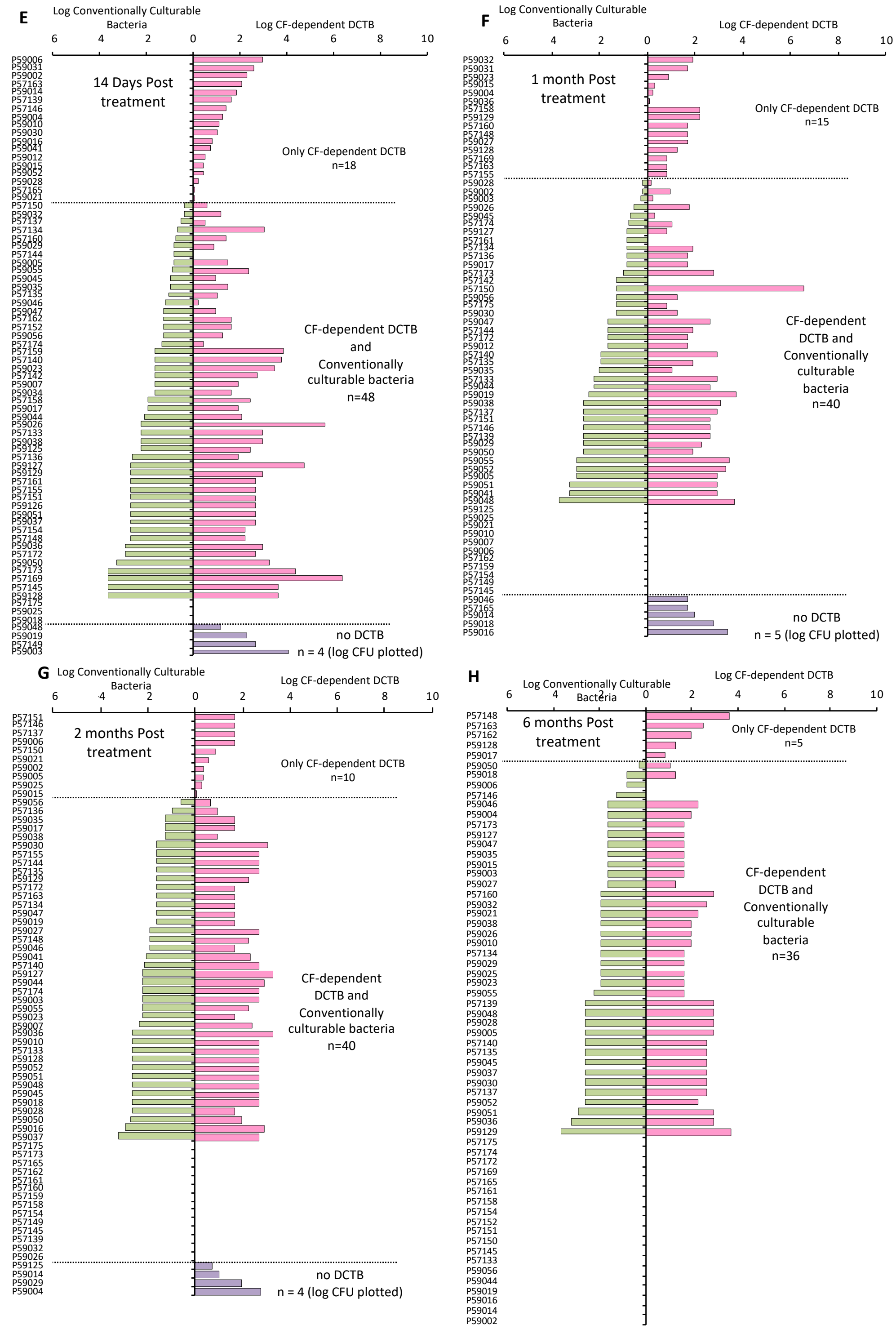

Supplementary Figure 5: Changes in DCTB populations during treatment. (A) Schematic of the LLD and CFU assays for detection of culturable and differentially culturable bacterial. CF-dependent (pink) and CF-independent (green) DCTB in sputum are detected. For sputum samples with no DCTB, CFU counts were plotted as it represents the highest bacterial count noted. For patients with no MPN and CFU counts (0), there are no bars on the plots. (B, C, D, E, F, G and H) Represent enrolment and days 3, 7, 14, 1 month, 2 months and 6 months post treatment respectively.

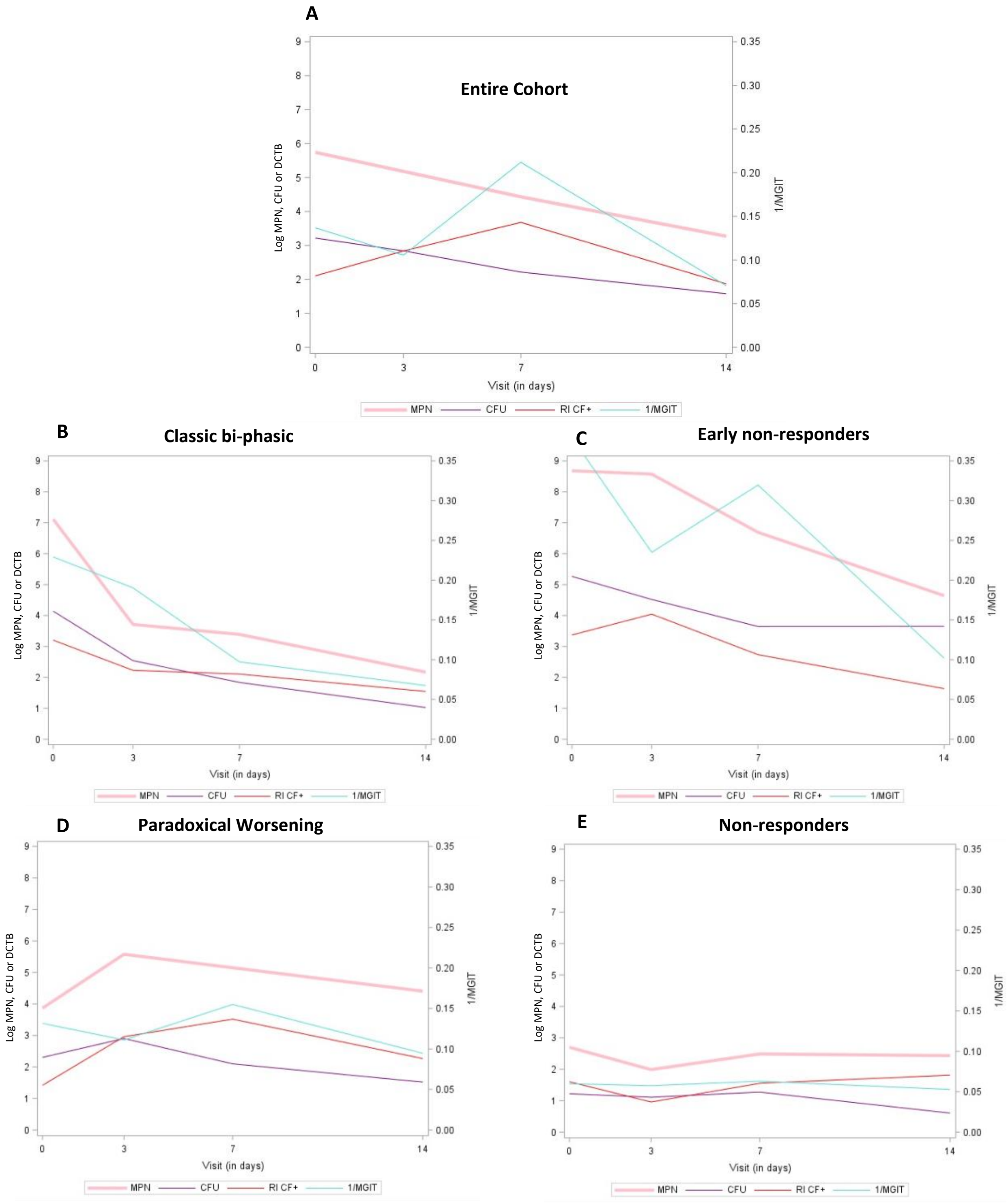

Supplementary Figure 6. Linear Mixed Effects modelling on bacterial clearance using different assays. (A) LOESS modelling over time for the combined cohort and (B-E) for the subcategories including Classic bi-phasic, Early non-responders, Paradoxical worsening, and Non–responsive groups, based on the median trend line of the MPN derived from LLDs. For each individual graph, slopes were compared to determine differences in the rates of decline (reported in Supplementary Table 6)

A

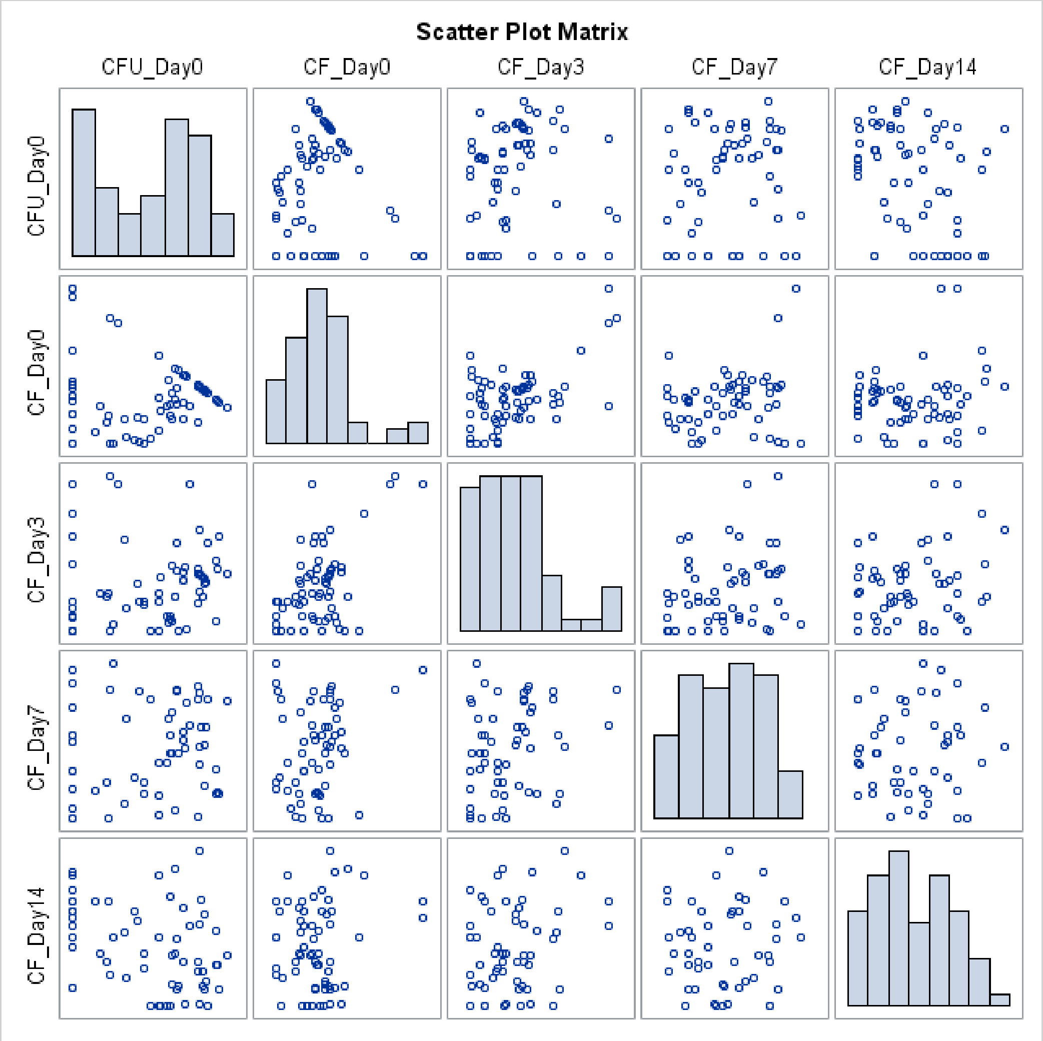

Pearson Correlation Coefficients
Prob > |r| under H0: Rho=0
Number of Observations

	CFU_Day0	CF_Day0	CF_Day3	CF_Day7	CF_Day14
CFU_Day0	1.00000	-0.07097	0.10783	0.09345	-0.35133
	-	0.5536	0.3888	0.4814	0.0051
	72	72	66	59	62
CF_Day0	-0.07097	1.00000	0.54778	0.22380	0.13270
	0.5536	-	<.0001	0.0884	0.3039
	72	72	66	59	62
CF_Day3	0.10783	0.54778	1.00000	0.24365	0.23333
	0.3888	<.0001	-	0.0730	0.0807
	66	66	66	55	57
CF_Day7	0.09345	0.22380	0.24365	1.00000	0.01579
	0.4814	0.0884	0.0730	-	0.9124
	59	59	55	59	51
CF_Day14	-0.35133	0.13270	0.23333	0.01579	1.00000
	0.0051	0.3039	0.0807	0.9124	-
	62	62	57	51	63

Supplementary Figure 7A: Correlation between CFU at Day 0 versus CF-Supplemented MPNs (CF_) measures at Day 0, 3, 7 and 14

B

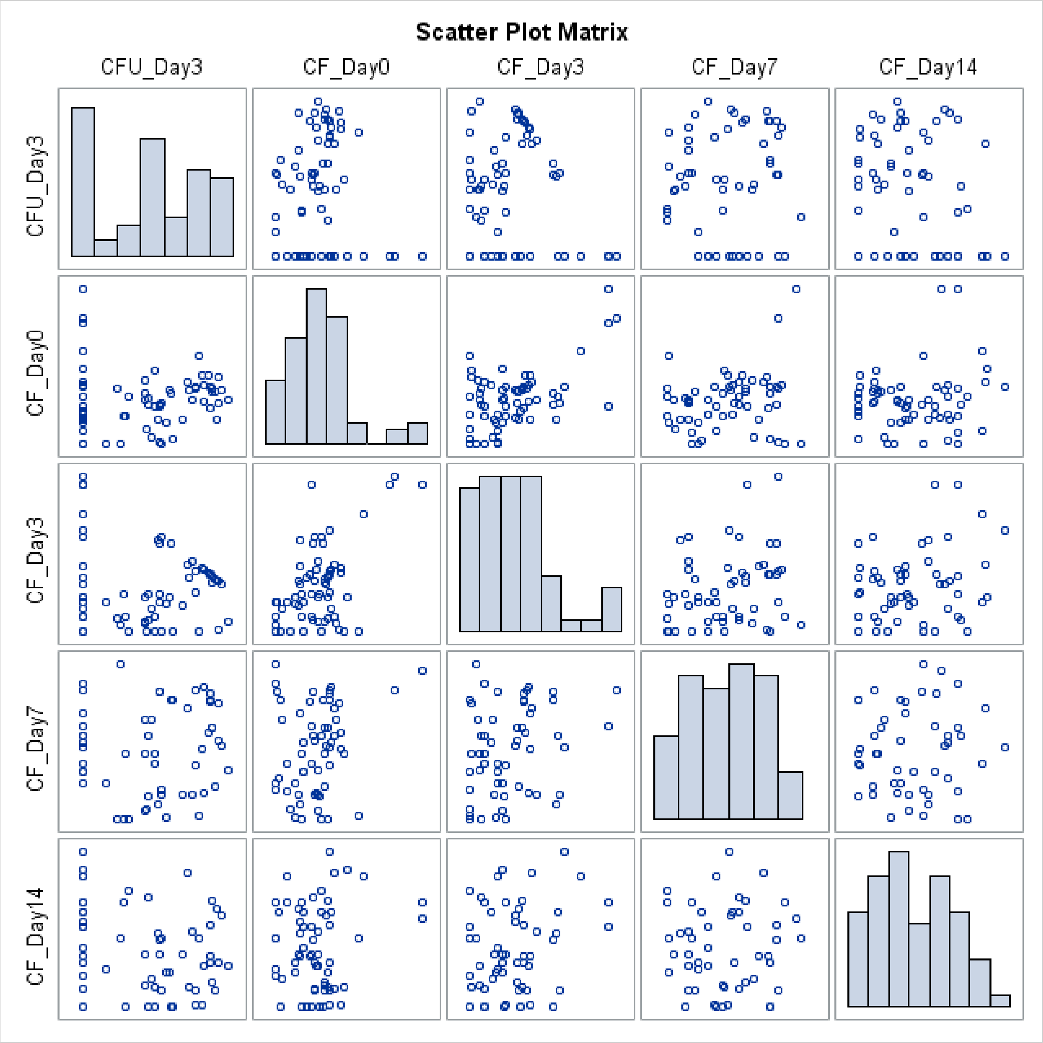

Pearson Correlation Coefficients<br>Prob > |r| under H0: Rho=0<br>Number of Observations					
	CFU_Day3	CF_Day0	CF_Day3	CF_Day7	CF_Day14
CFU_Day3	1.00000	0.01044	-0.15415	0.06872	-0.30913
	-	0.9337	0.2165	0.6181	0.0193
	66	66	66	55	57
CF_Day0	0.01044	1.00000	0.54778	0.22380	0.13270
	0.9337	-	<.0001	0.0884	0.3039
	66	72	66	59	62
CF_Day3	-0.15415	0.54778	1.00000	0.24365	0.23333
	0.2165	<.0001	-	0.0730	0.0807
	66	66	66	55	57
CF_Day7	0.06872	0.22380	0.24365	1.00000	0.01579
	0.6181	0.0884	0.0730	-	0.9124
	55	59	55	59	51
CF_Day14	-0.30913	0.13270	0.23333	0.01579	1.00000
	0.0193	0.3039	0.0807	0.9124	-
	57	62	57	51	63

Supplementary Figure 7B: Correlation between CFU at Day 3 versus CF-Supplemented MPNs (CF_) measures at Day 0, 3, 7 and 14

C

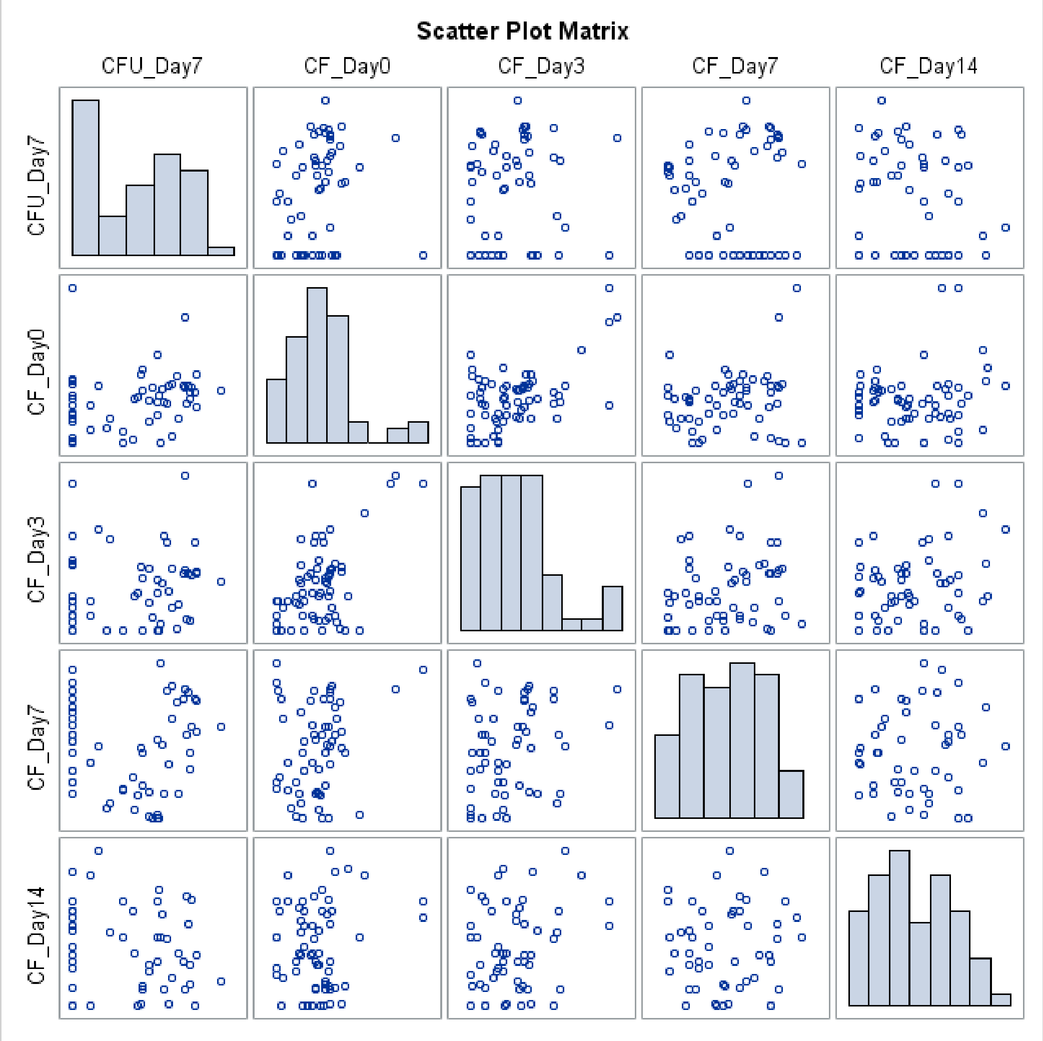

Pearson Correlation Coefficients<br>Prob > |r| under H0: Rho=0<br>Number of Observations					
	CFU_Day7	CF_Day0	CF_Day3	CF_Day7	CF_Day14
CFU_Day7	1.00000	0.22302	0.08992	0.06083	-0.21876
	-	0.0895	0.5138	0.6472	0.1230
	59	59	55	59	51
CF_Day0	0.22302	1.00000	0.54778	0.22380	0.13270
	0.0895	-	<.0001	0.0884	0.3039
	59	72	66	59	62
CF_Day3	0.08992	0.54778	1.00000	0.24365	0.23333
	0.5138	<.0001	-	0.0730	0.0807
	55	66	66	55	57
CF_Day7	0.06083	0.22380	0.24365	1.00000	0.01579
	0.6472	0.0884	0.0730	-	0.9124
	59	59	55	59	51
CF_Day14	-0.21876	0.13270	0.23333	0.01579	1.00000
	0.1230	0.3039	0.0807	0.9124	-
	51	62	57	51	63

Supplementary Figure 7C: Correlation between CFU at Day 7 versus CF-Supplemented MPNs (CF_) measures at Day 0, 3, 7 and 14

D

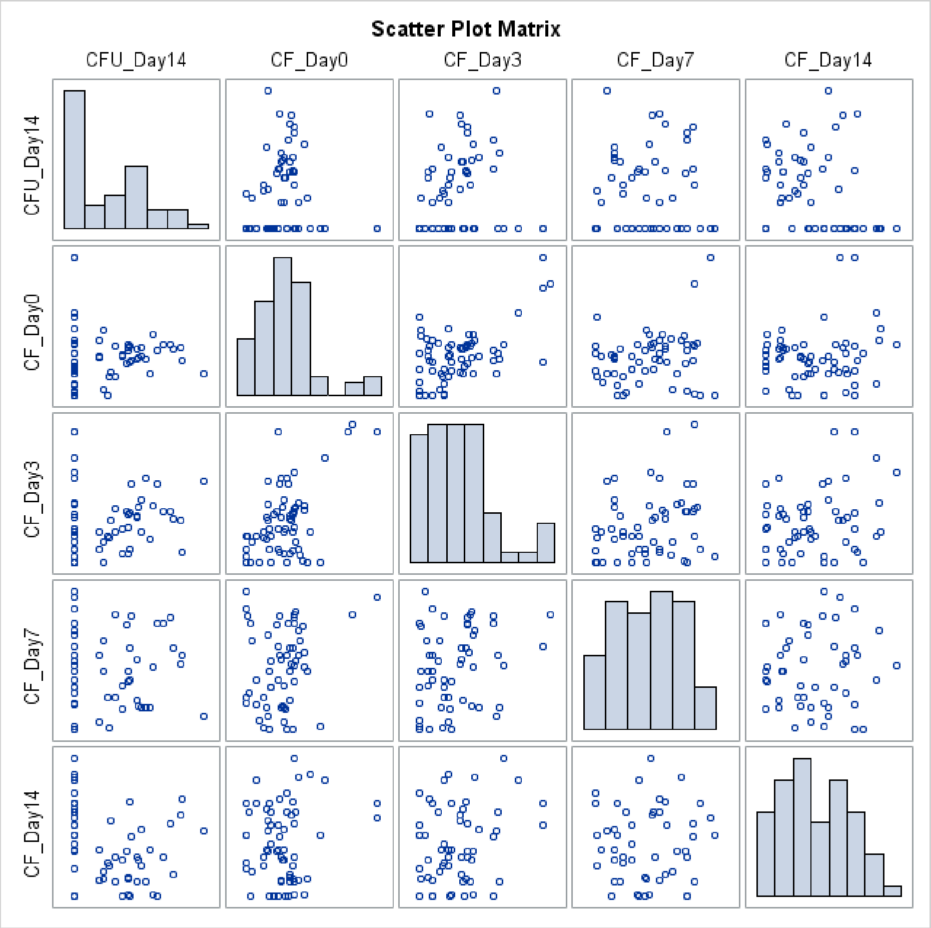

Table 4: Pearson Correlation Coefficients<br>Prob > |r| under H0: Rho=0<br>Number of Observations					
	CFU_Day14	CF_Day0	CF_Day3	CF_Day7	CF_Day14
CFU_Day14	1.00000	0.05826	0.13689	-0.00552	-0.34201
	-	0.6529	0.3099	0.9694	0.0061
	63	62	57	51	63
CF_Day0	0.05826	1.00000	0.54778	0.22380	0.13270
	0.6529	-	<.0001	0.0884	0.3039
	62	72	66	59	62
CF_Day3	0.13689	0.54778	1.00000	0.24365	0.23333
	0.3099	<.0001	-	0.0730	0.0807
	57	66	66	55	57
CF_Day7	-0.00552	0.22380	0.24365	1.00000	0.01579
	0.9694	0.0884	0.0730	-	0.9124
	51	59	55	59	51
CF_Day14	-0.34201	0.13270	0.23333	0.01579	1.00000
	0.0061	0.3039	0.0807	0.9124	-
	63	62	57	51	63

Supplementary Figure 7D: Correlation between CFU at Day 14 versus CF-Supplemented MPNs (CF_) measures at Day 0, 3, 7 and 14

Supplementary Figure 7. Correlation analysis. Panels A-C depict correlation analysis between CFUs and CF-Supplemented MPN assays (shown as CF_) for all participants at days 0, 3, 7, and 14. Tables depict correlation coefficients.

Participants with no CFUs (or 1 positive CFU) during the first month of treatment

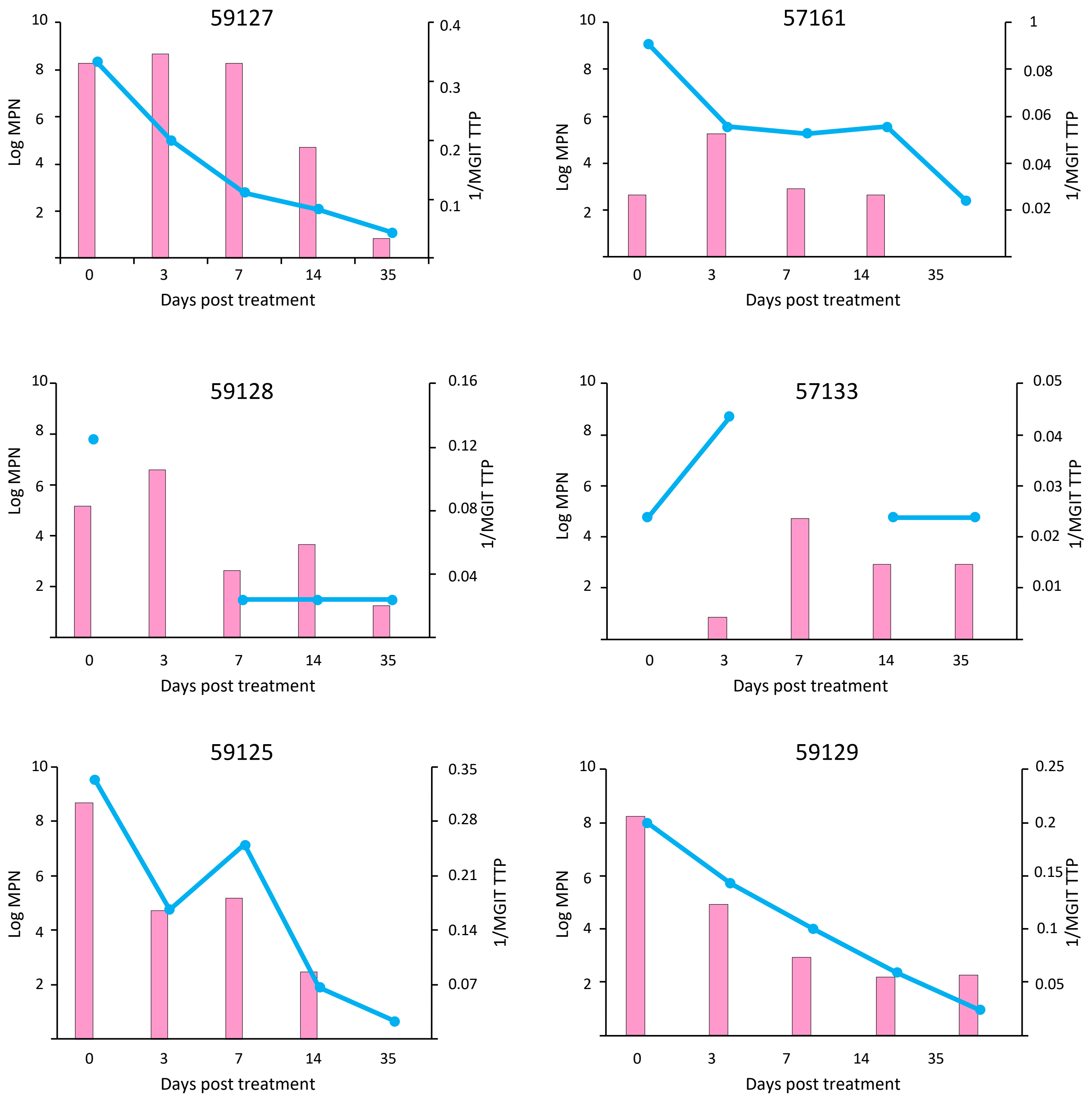

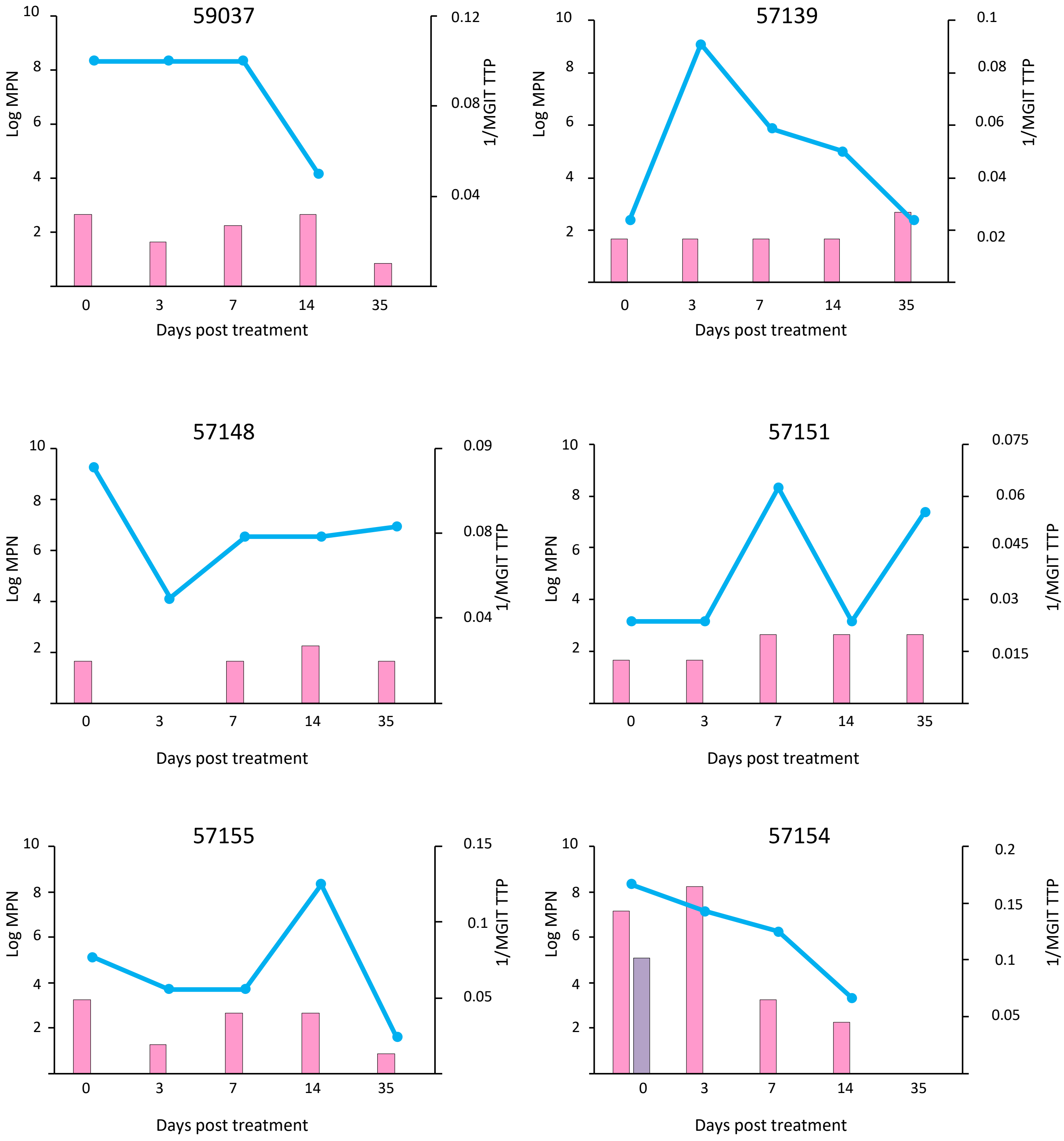
59037
Log MPN
1/MGIT TTP
10
8
6
4
2
0.12
0.08
0.04
0
3
7
14
35
Days post treatment
57139
Log MPN
1/MGIT TTP
10
8
6
4
2
0.1
0.08
0.06
0.04
0.02
0
3
7
14
35
Days post treatment
57148
Log MPN
1/MGIT TTP
10
8
6
4
2
0.09
0.08
0.04
0
3
7
14
35
Days post treatment
57151
Log MPN
1/MGIT TTP
10
8
6
4
2
0.075
0.06
0.045
0.03
0.015
0
3
7
14
35
Days post treatment
57155
Log MPN
1/MGIT TTP
10
8
6
4
2
0.15
0.1
0.05
0
3
7
14
35
Days post treatment
57154
Log MPN
1/MGIT TTP
10
8
6
4
2
0.2
0.15
0.1
0.05
0
3
7
14
35
Days post treatment

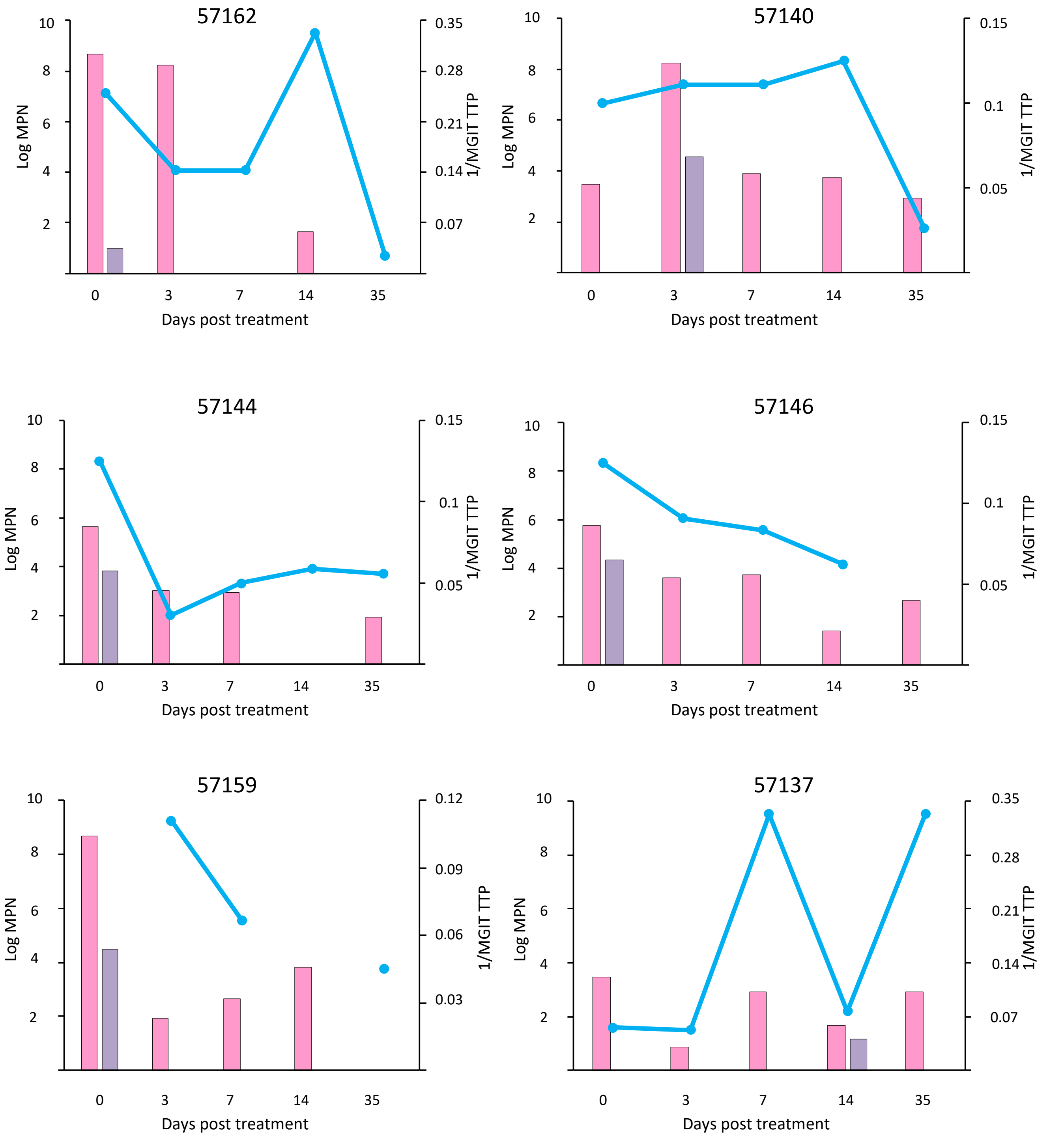

Supplementary Figure 8. Individual patient graphs for participants with no CFU or one positive CFU during the 5 sampling time points of the first month of treatment. Each panel represents individual participants with no CFU's or 1 recorded CFU value across the sampling time points during the first month of treatment. Pink bars represent MPN values, purple bars CFU values and the blue line represents 1/Mycobacterial Growth Indicator Tube Time to Positivity (MGIT TTP), gaps in the line represent contaminated MGITs.

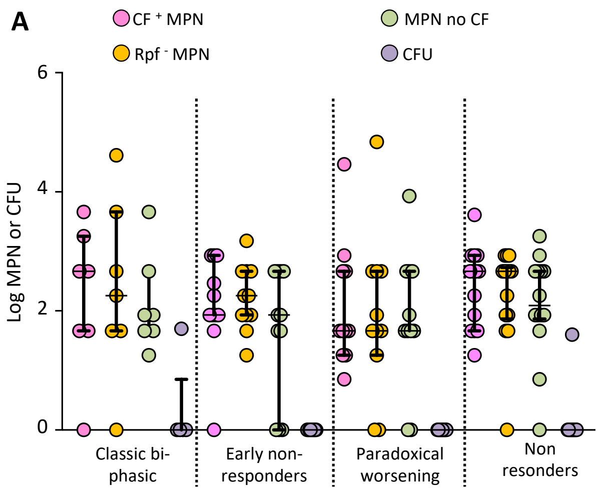

Supplementary Figure 9: Residual DCTB analysis after treatment completion. Shown are residual MPNs and CFUs in treatment response categories. There were no significant differences in MPNs within and between categories

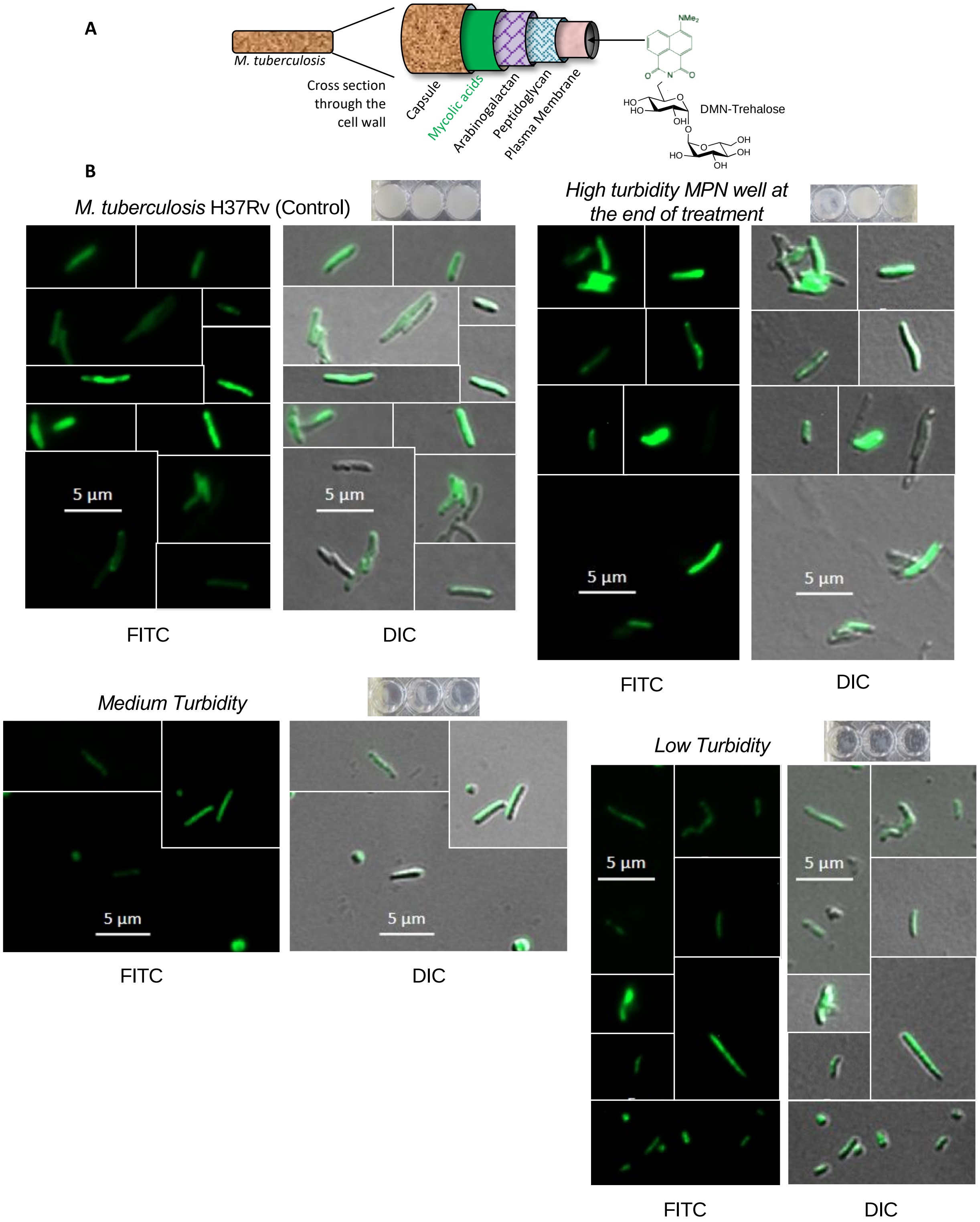

Supplementary Figure 10: Fluorescence microscopy of DMN-tre stained *M. tuberculosis* cultured from day 180 sputum samples in MPN plates. (A) Schematic representation of DMN-Tre staining mechanism. (B) Microscopy images. In each case, the left panel shows the fluorescence FITC signal and the right panel shows a merge of the DIC and FITC showing viable cells that take up the dye in the background of other cells sampled (Scale bar 5 µm). The *M. tuberculosis* control refers to an axenic culture of tubercle bacteria, which were stained and viewed alongside bacteria sampled from MPN assays. Pictorial representations of wells with high, medium and low turbidity are provided.

Supplementary Figure 11. Individual participant graphs. Each graph shows the outcome of each patient for the 5 parameters assessed (MPN, CF+) pink, (MPN Rpf-) orange (MPN No CF) green, (CFU) purple, (DCTB) black, (1/MGIT) turquoise, (1/Gene Xpert CT) brown. Error bars on MPN values represent the 95% confidence interval from the MPN calculator.

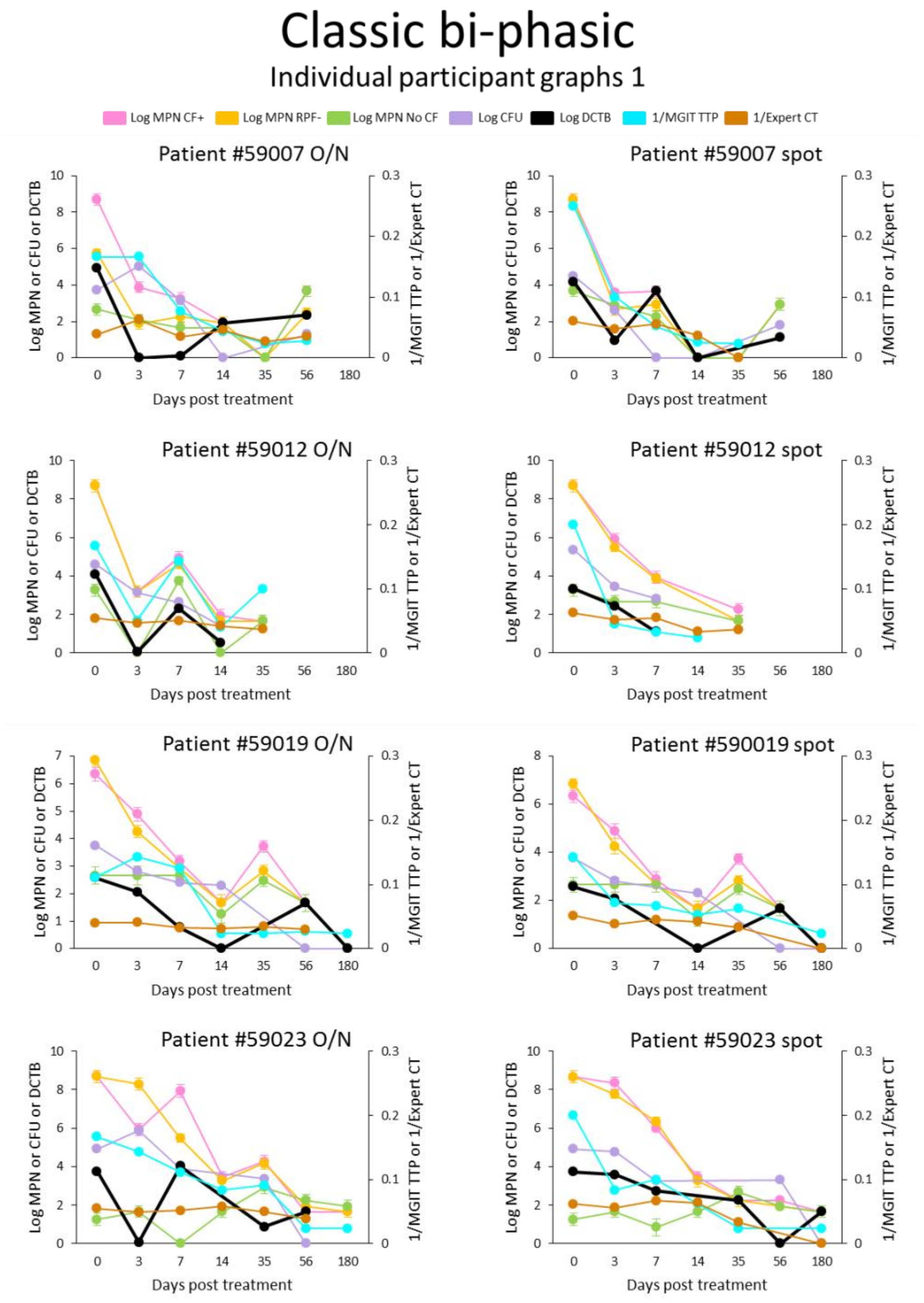

Classic bi-phasic

Individual participant graphs 2

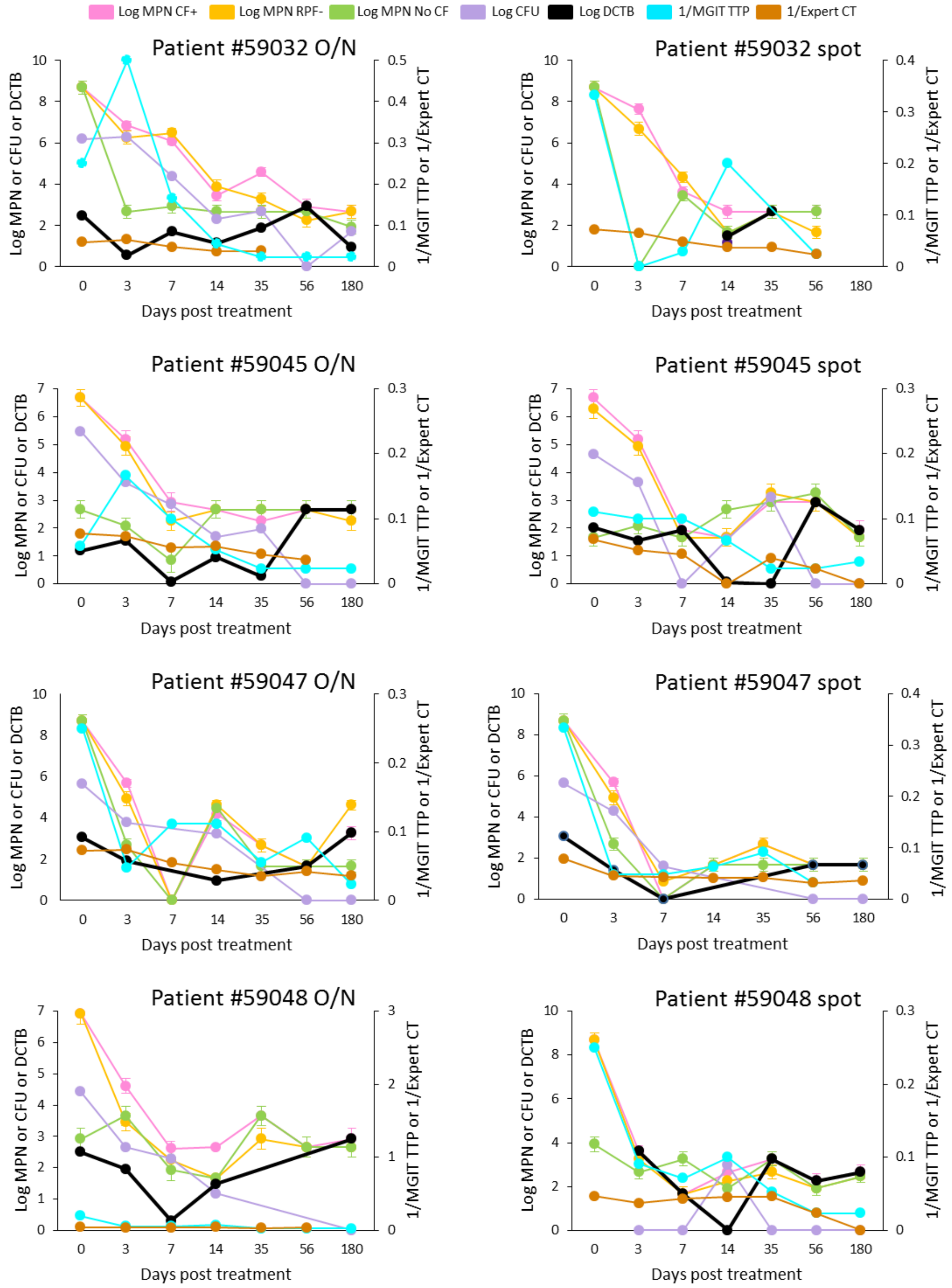

Classic bi-phasic

Individual participant graphs 3

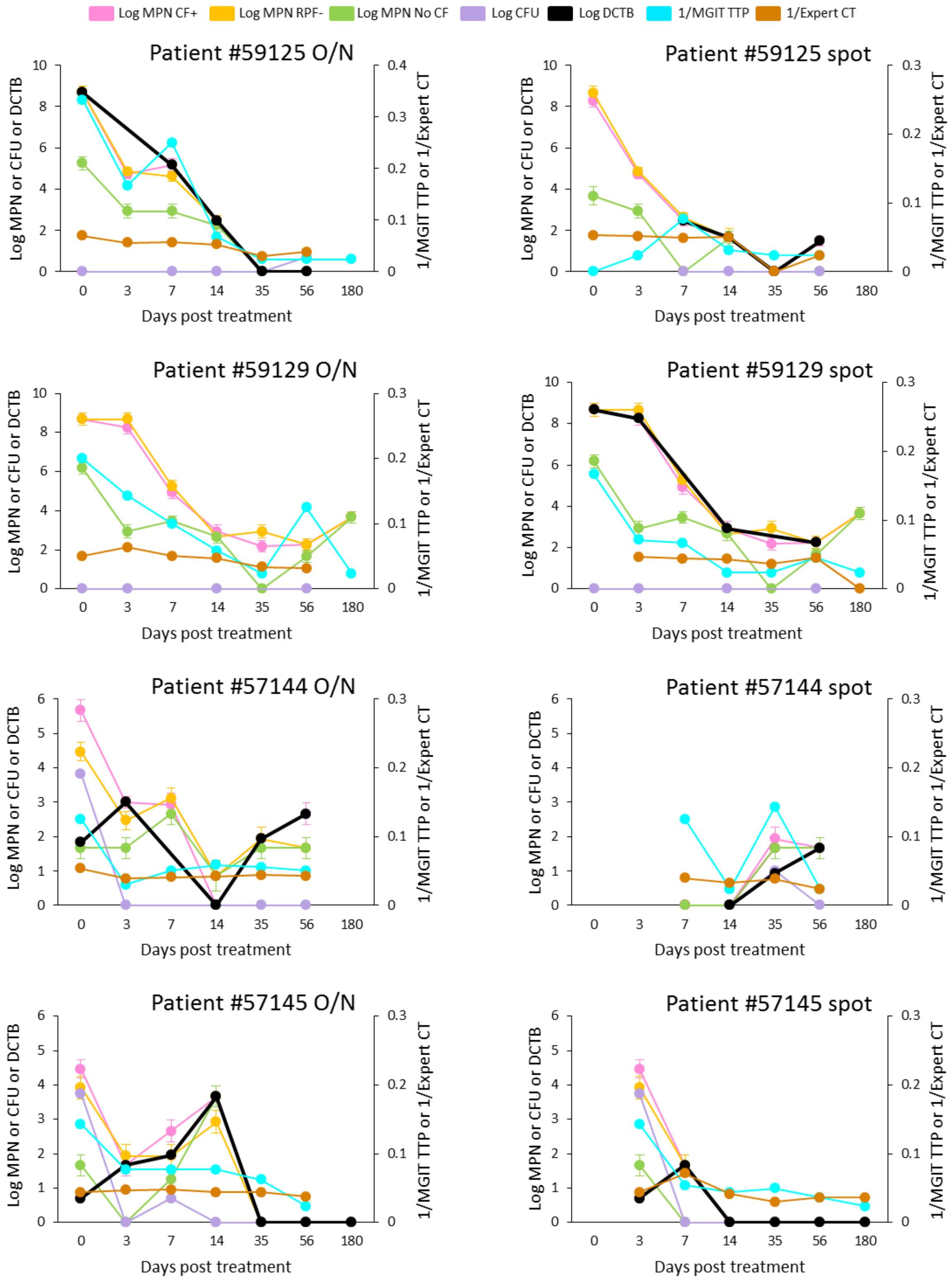

Classic bi-phasic

Individual participant graphs 4

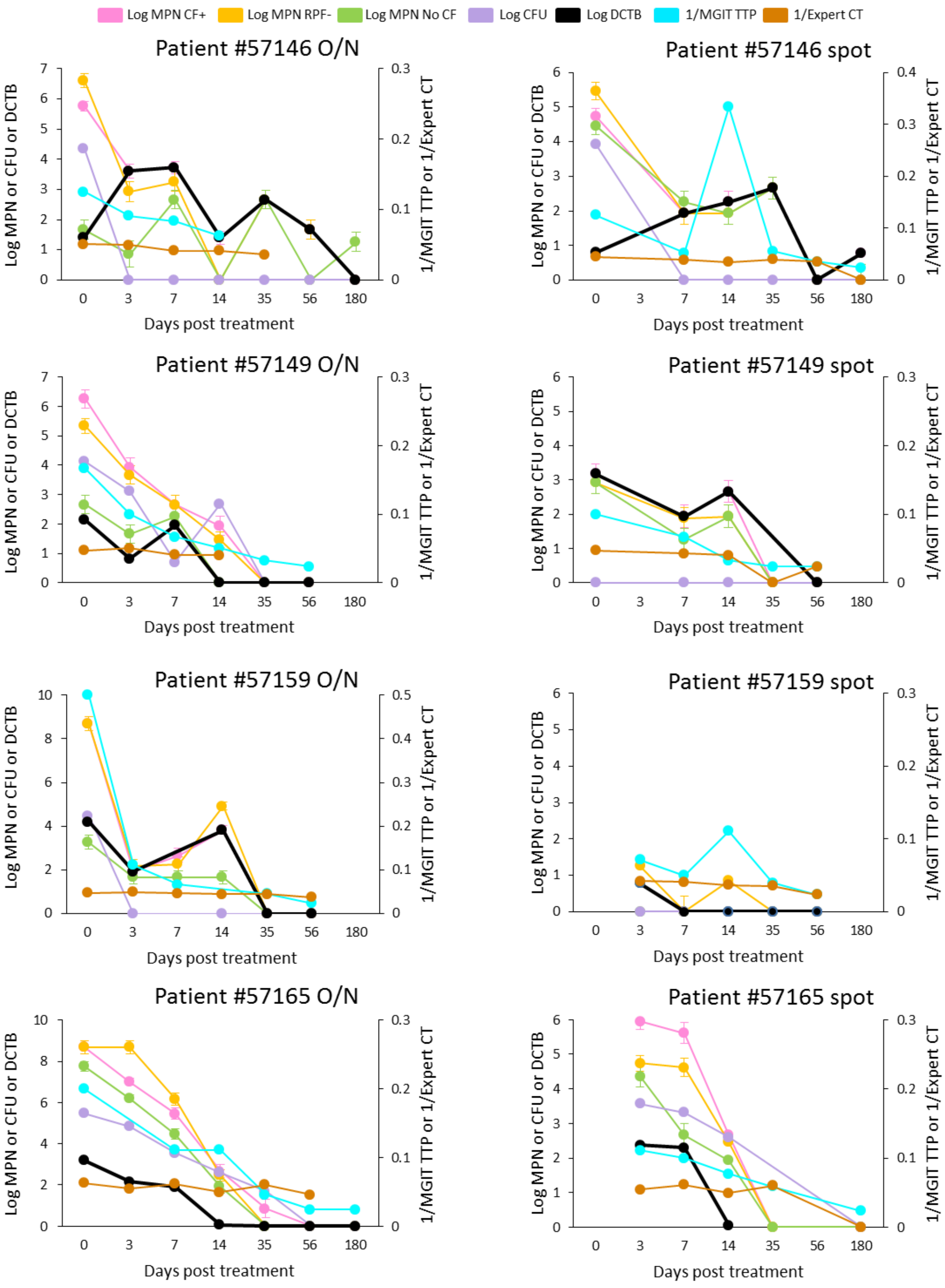

Classic bi-phasic

Individual participant graphs 5

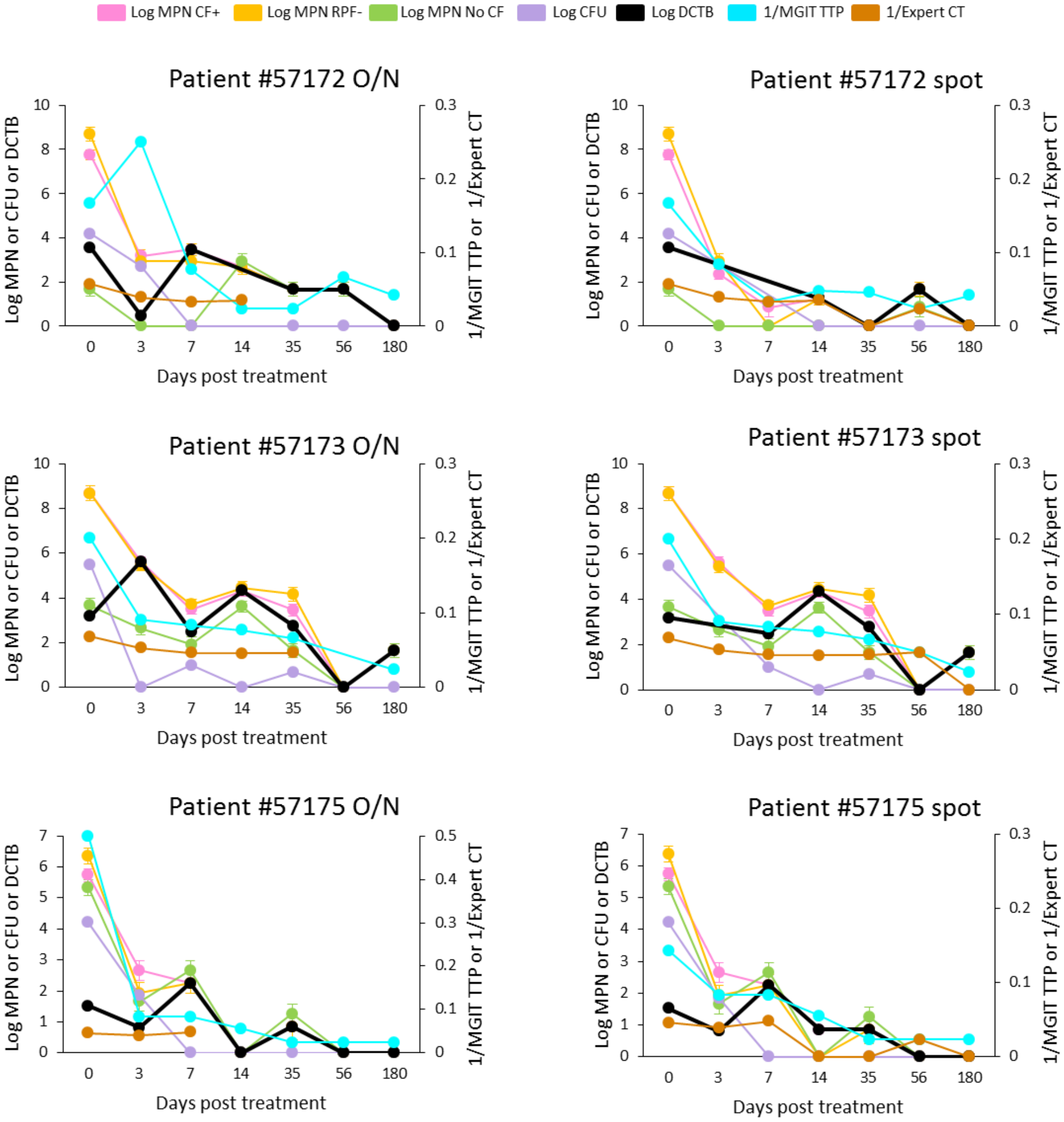

Early Non-responders

Individual participant graphs 1

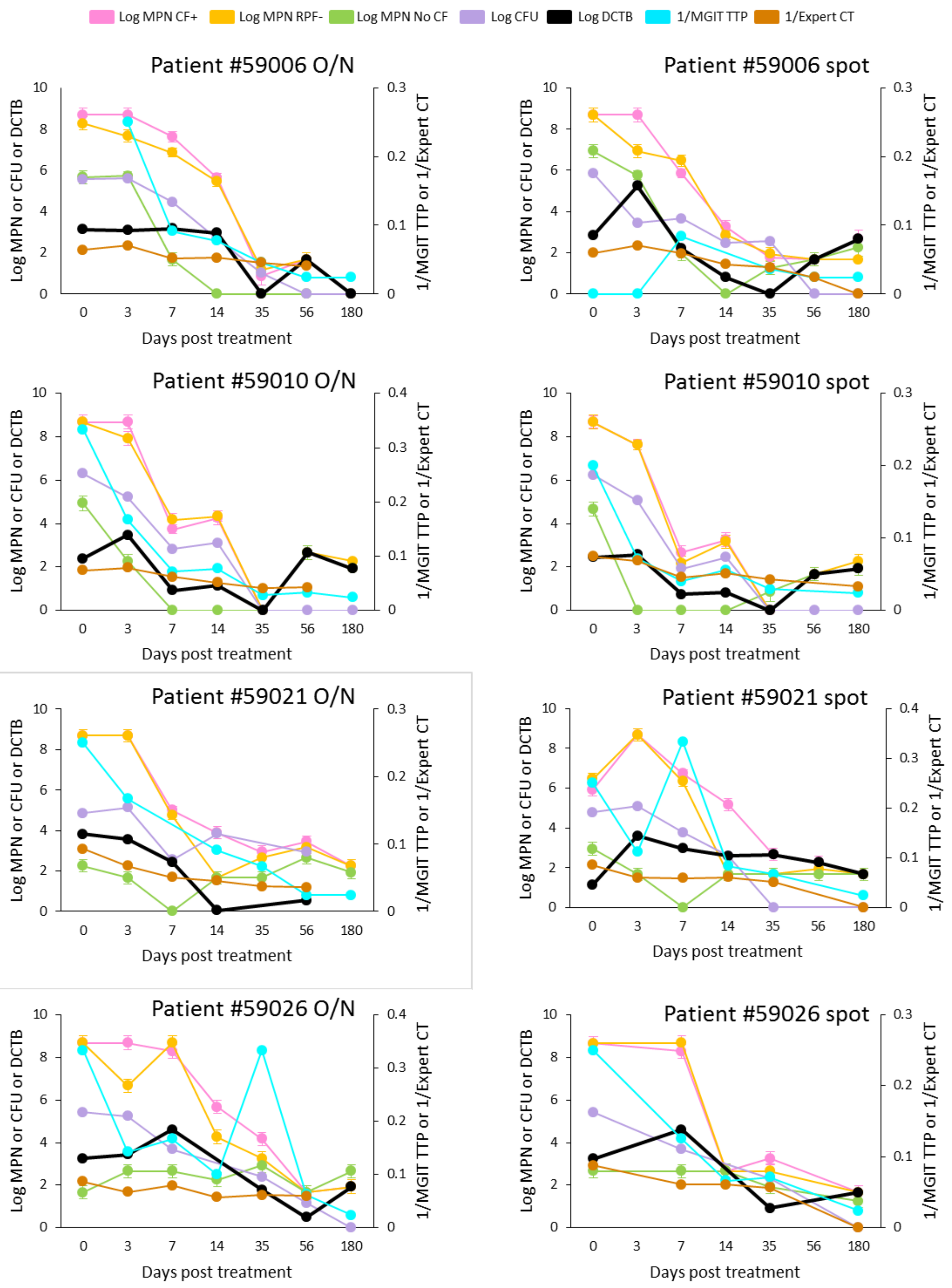

Early Non-responders

Individual participant graphs 2

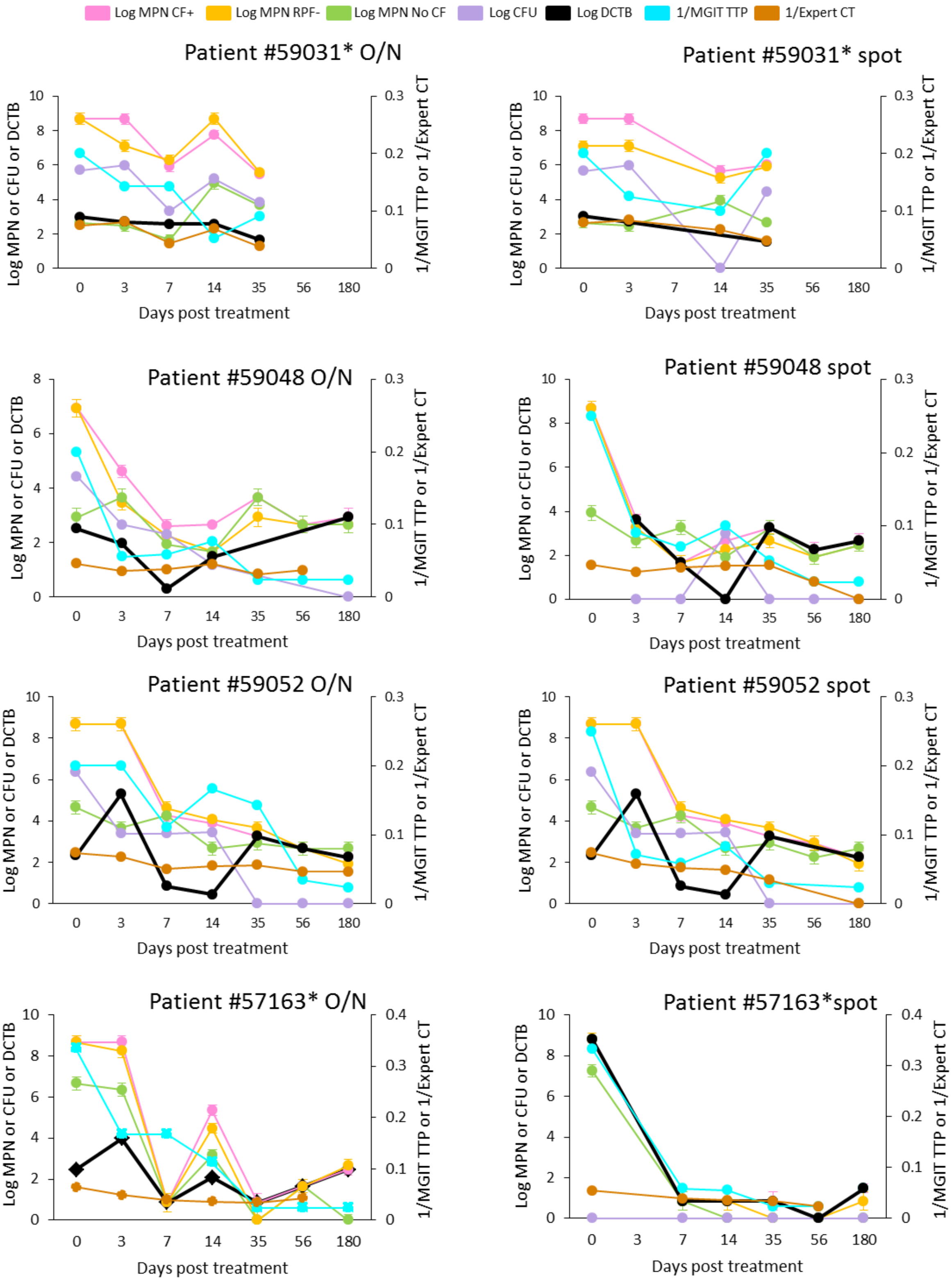

Early Non-responders

Individual participant graphs 3

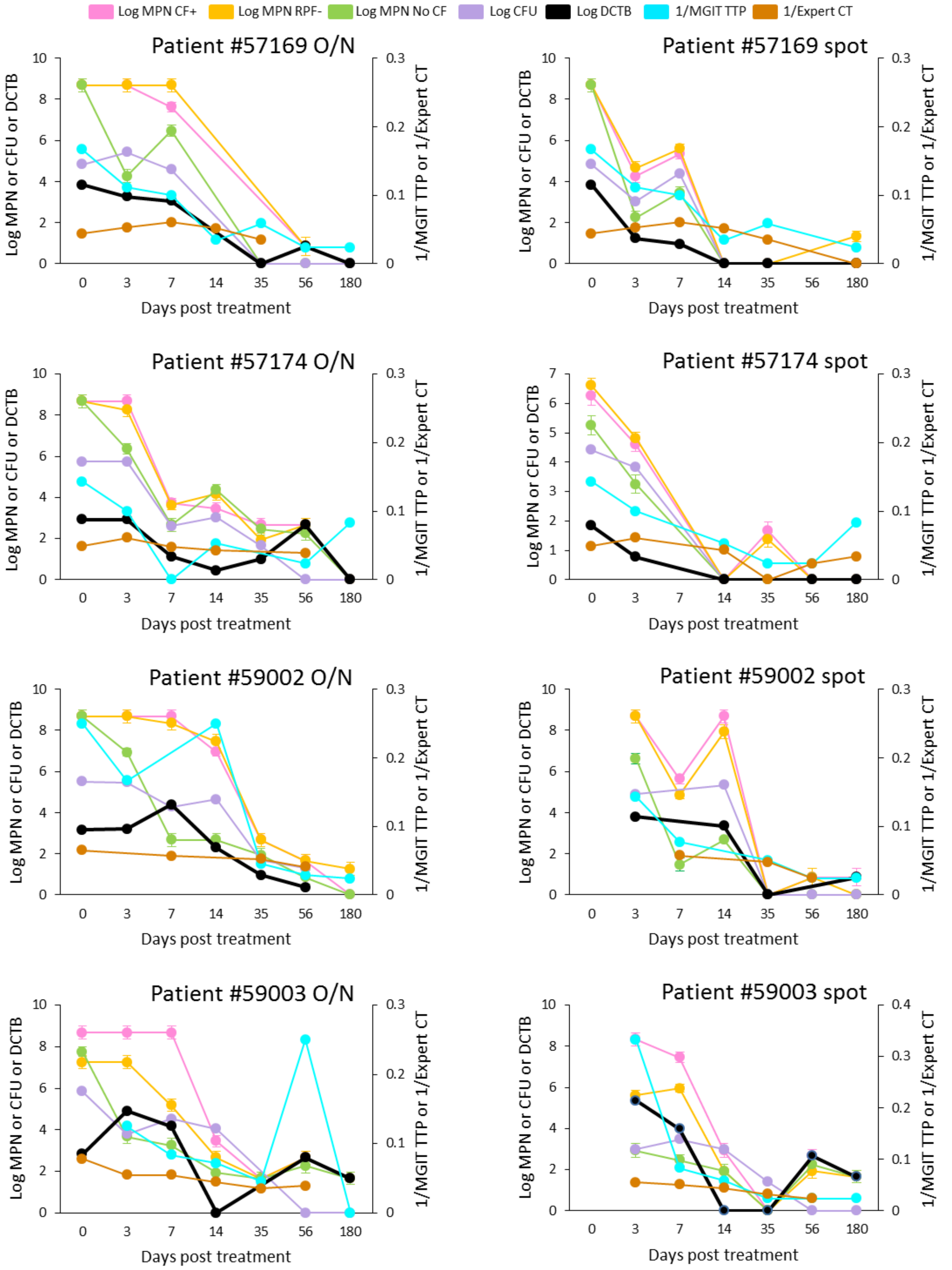

Early Non-responders

Individual participant graphs 4

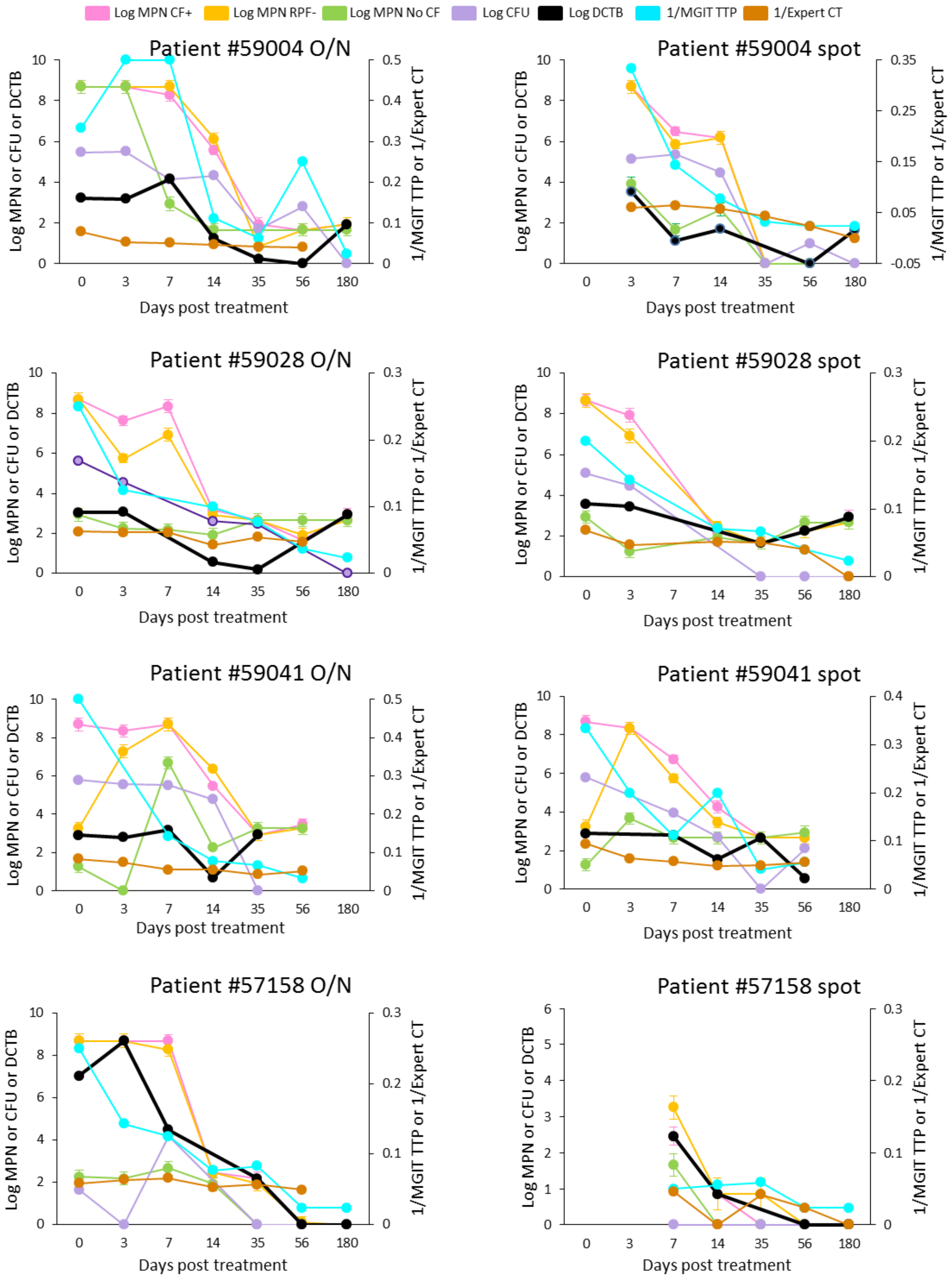

Early Non-responders

Individual participant graphs 5

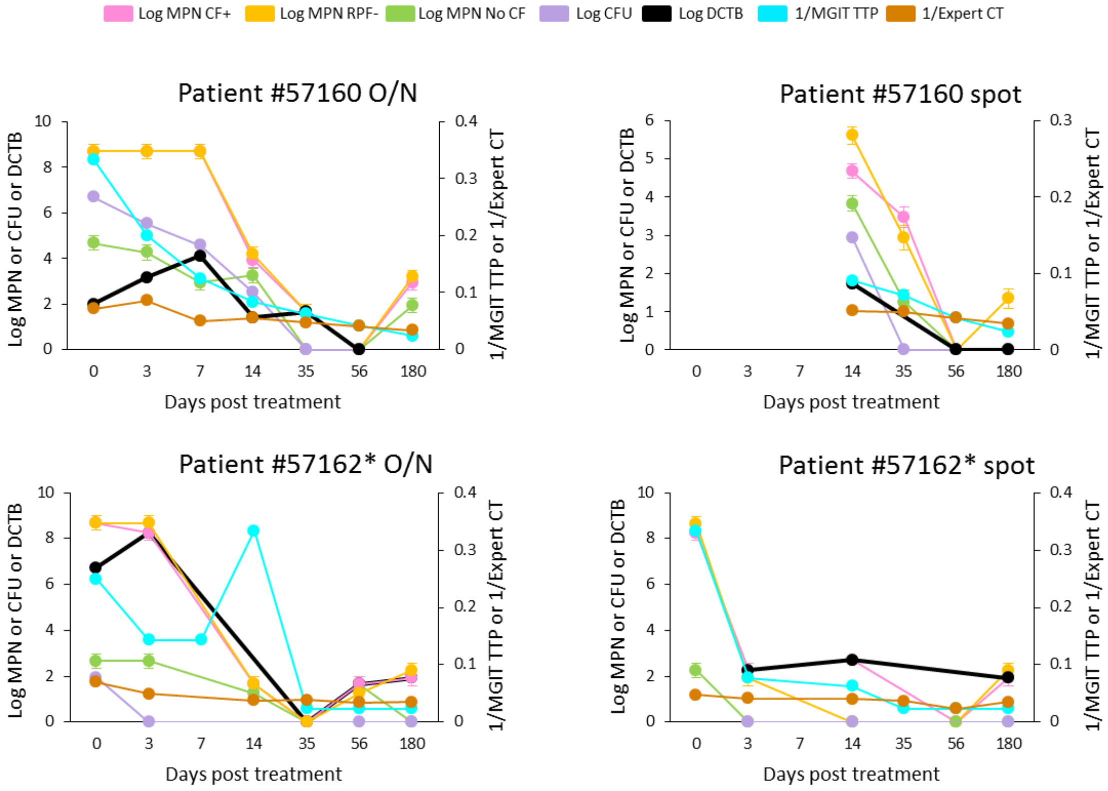

Paradoxical worsening

Individual participant graphs 1

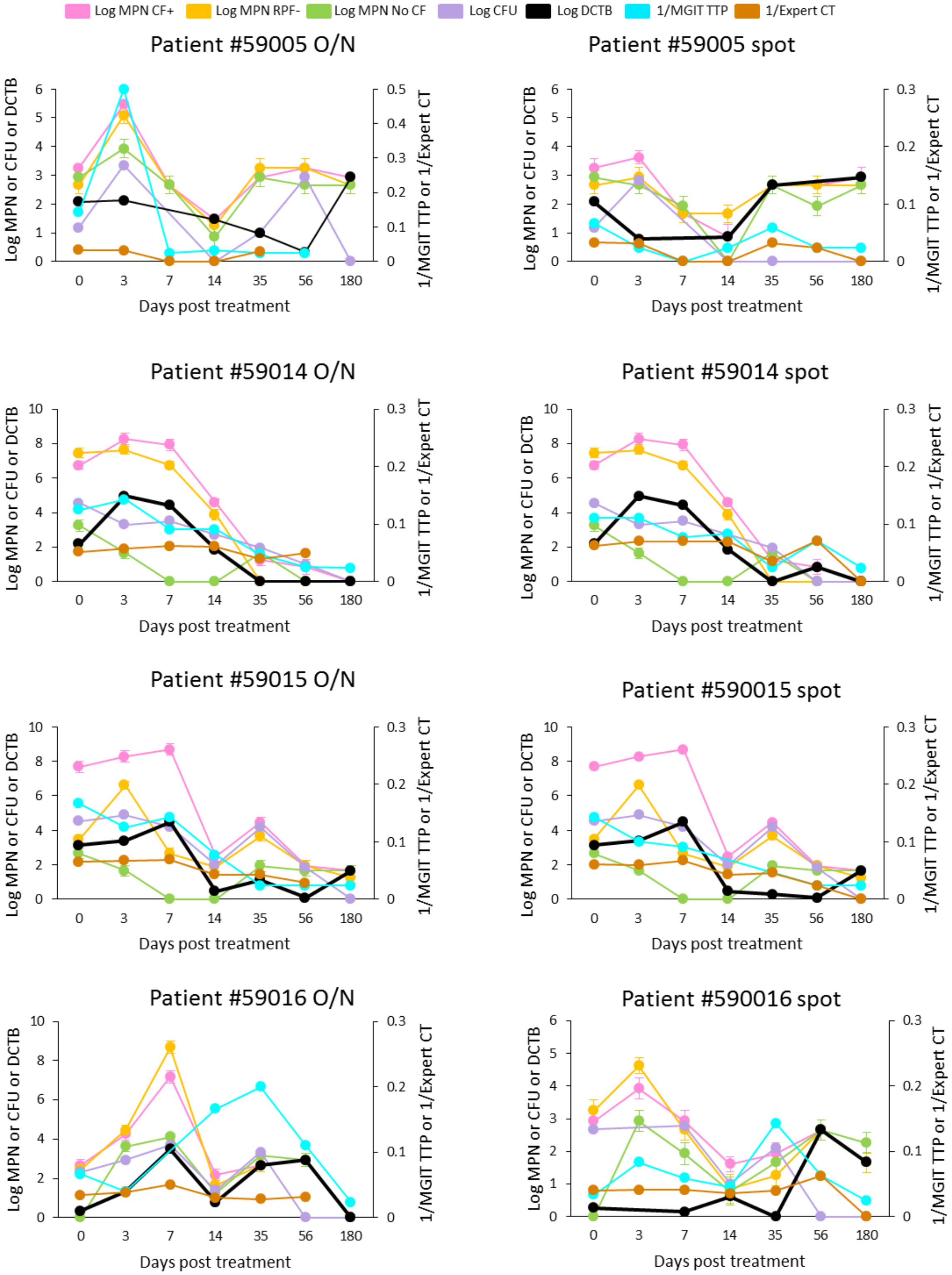

Paradoxical worsening

Individual participant graphs 2

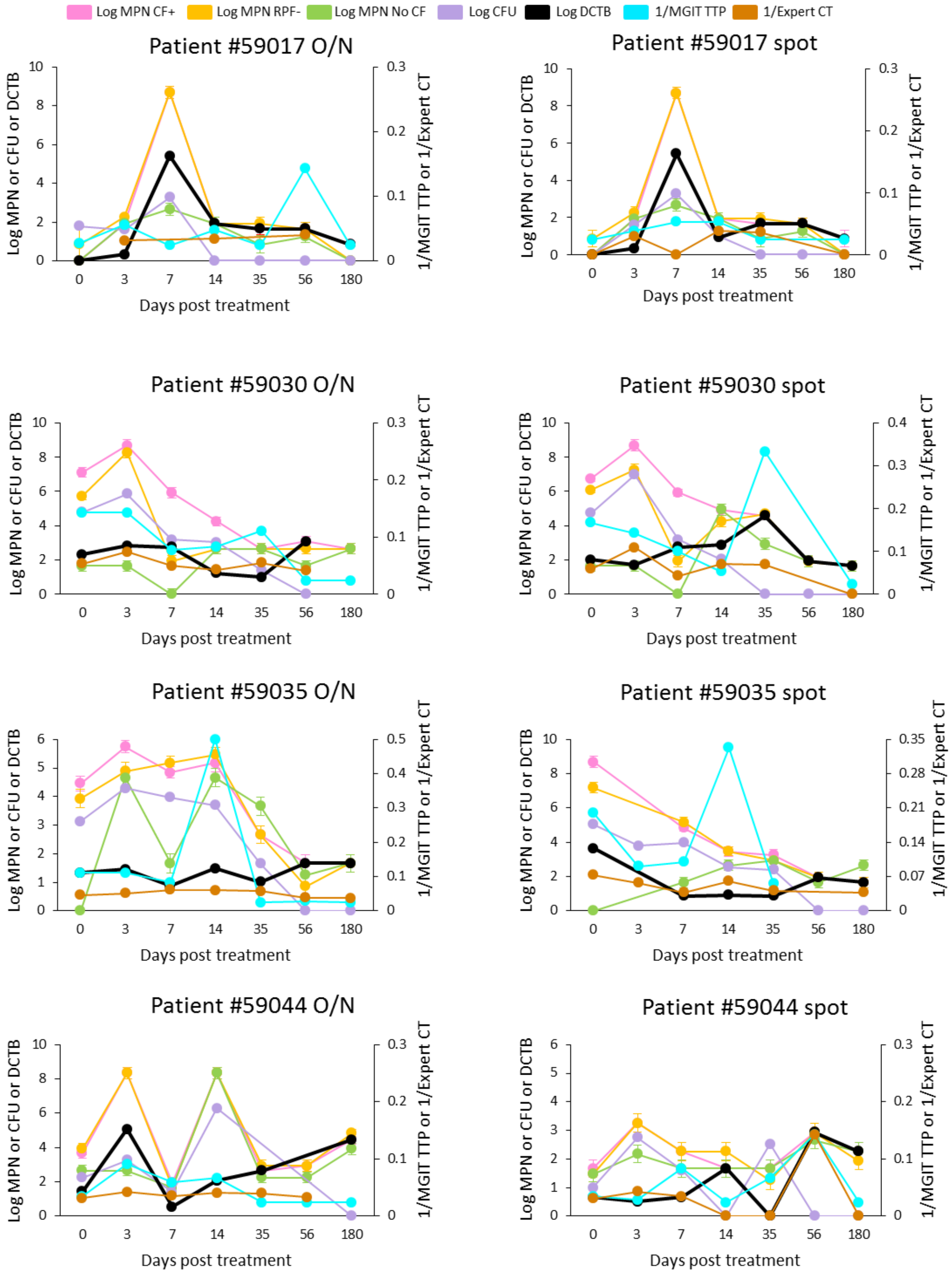

Paradoxical worsening

Individual participant graphs 3

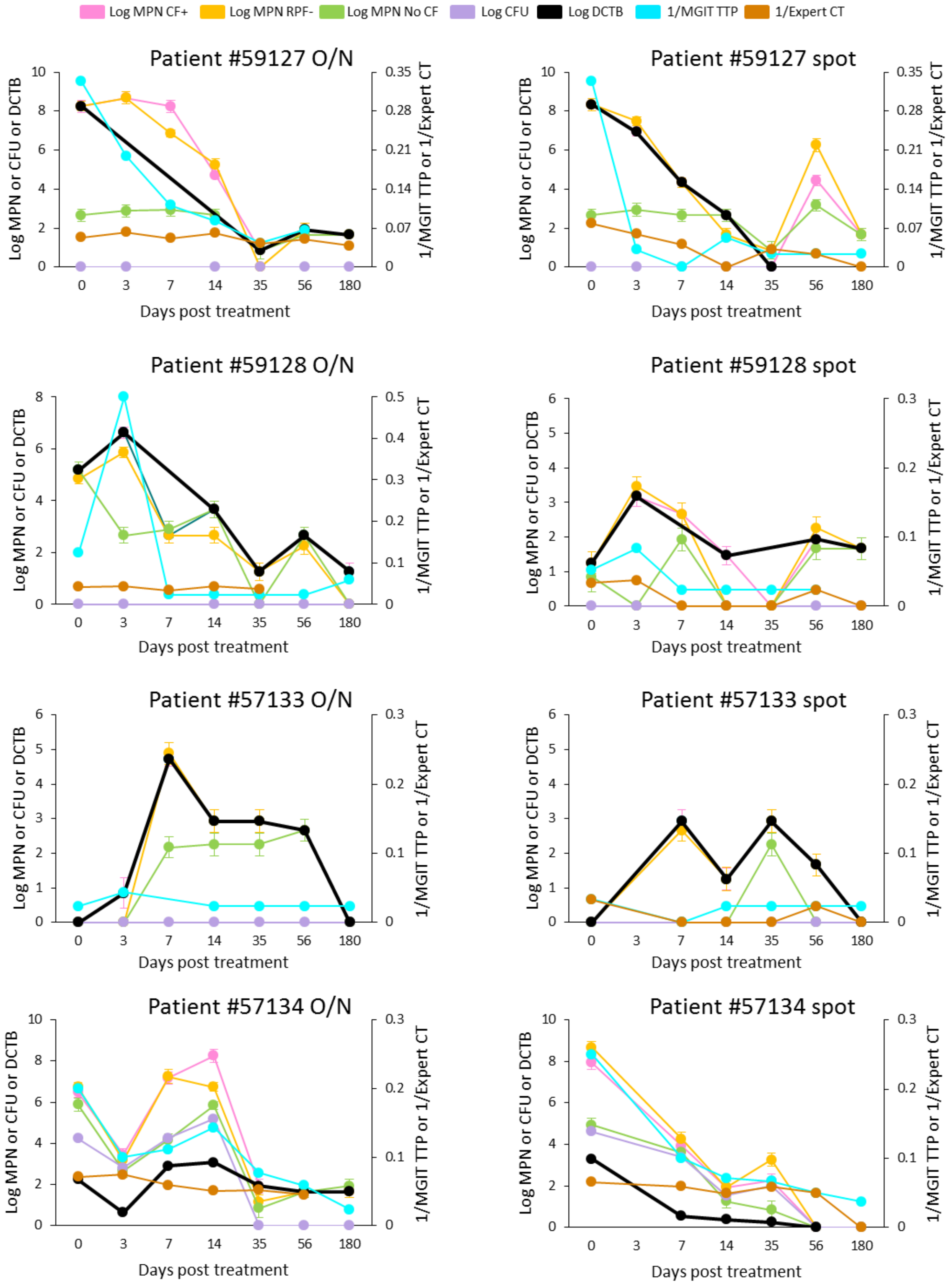

Paradoxical worsening

Individual participant graphs 4

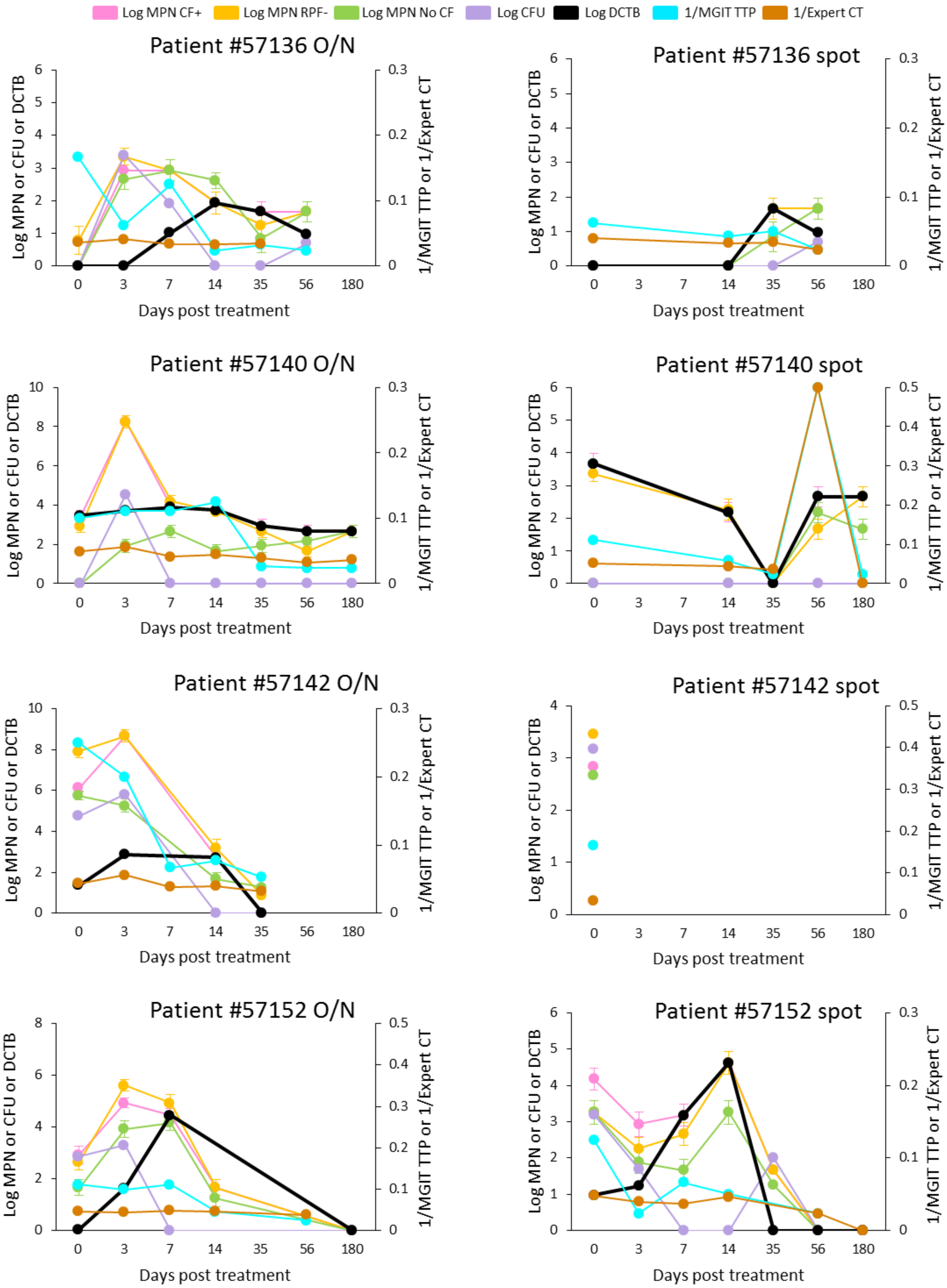

Paradoxical worsening

Individual participant graphs 5

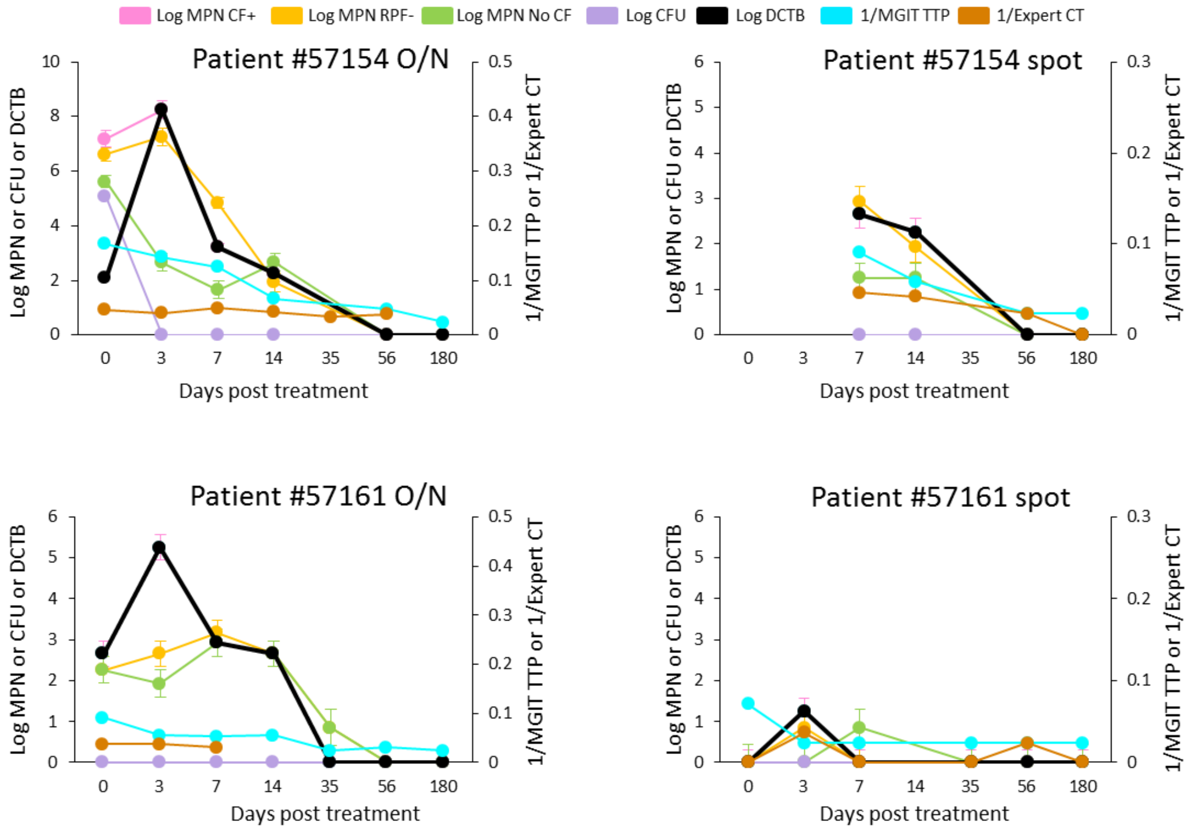

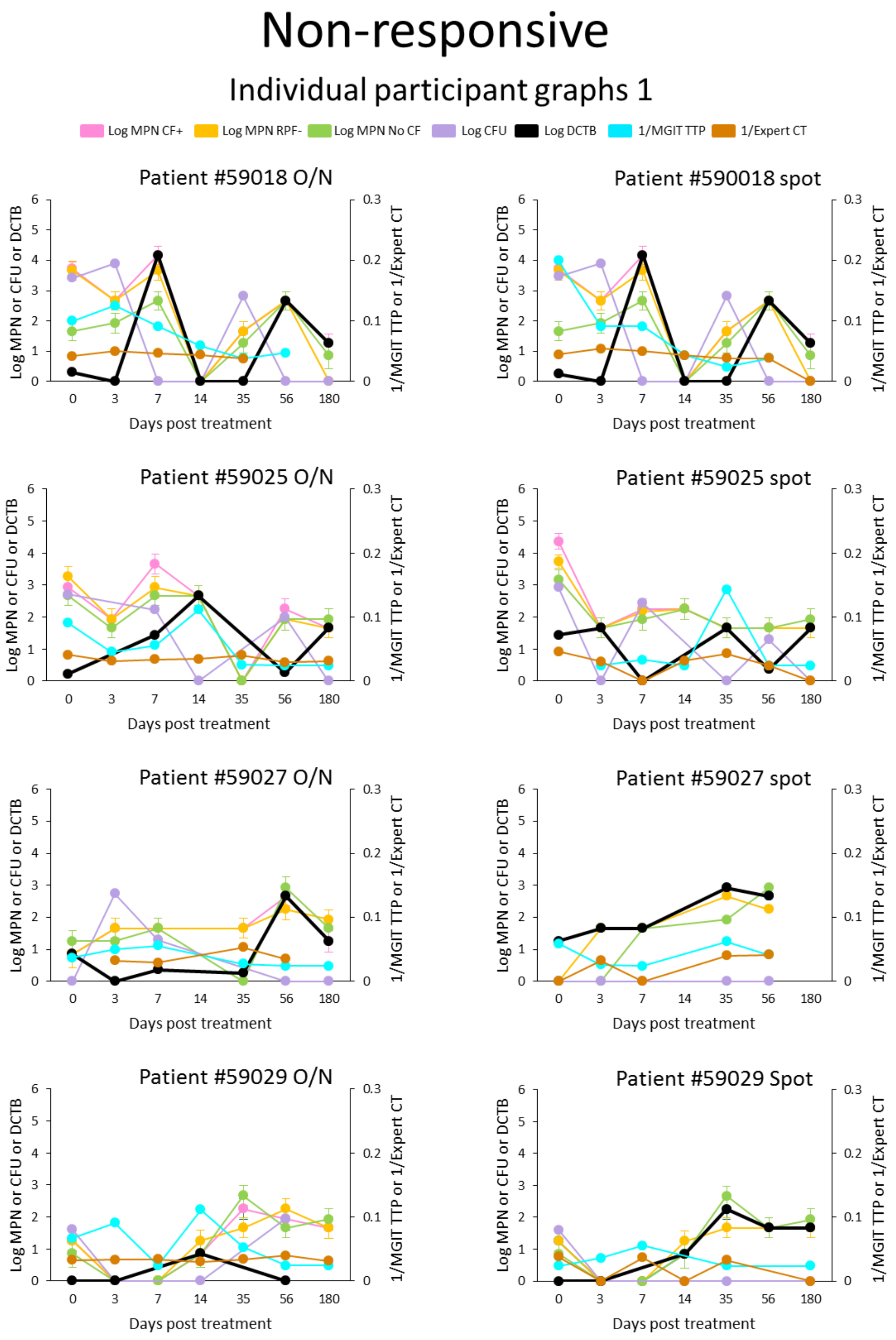
Non-responsive
Individual participant graphs 1
Log MPN CF+
Log MPN RPF-
Log MPN No CF
Log CFU
Log DCTB
1/MGIT TTP
1/Expert CT
Patient #59018 O/N
Patient #590018 spot
Patient #59025 O/N
Patient #59025 spot
Patient #59027 O/N
Patient #59027 spot
Patient #59029 O/N
Patient #59029 Spot
Log MPN or CFU or DCTB
1/MGIT TTP or 1/Expert CT
Days post treatment
0
3
7
14
35
56
180

Non-responsive

Individual participant graphs 2

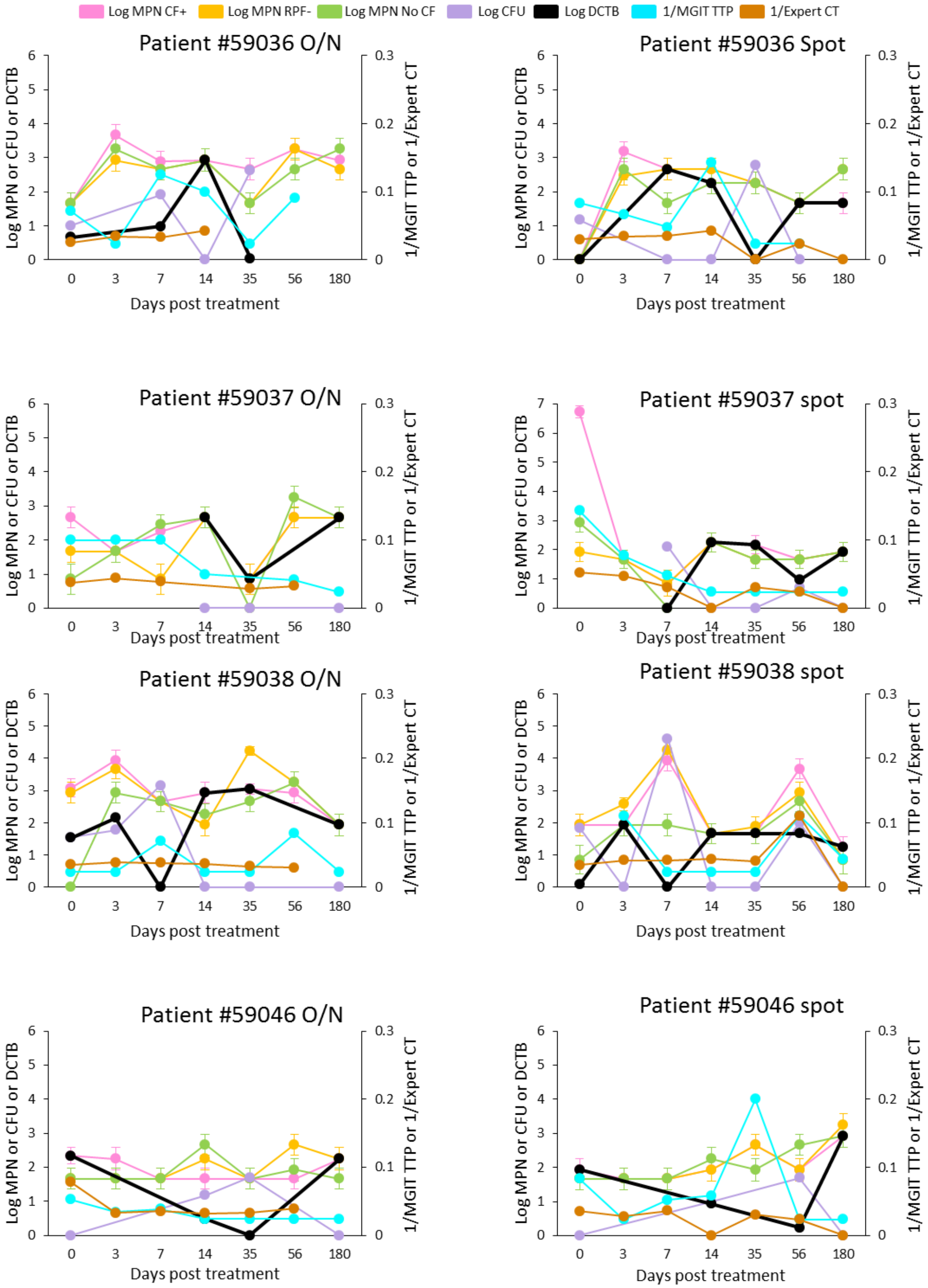

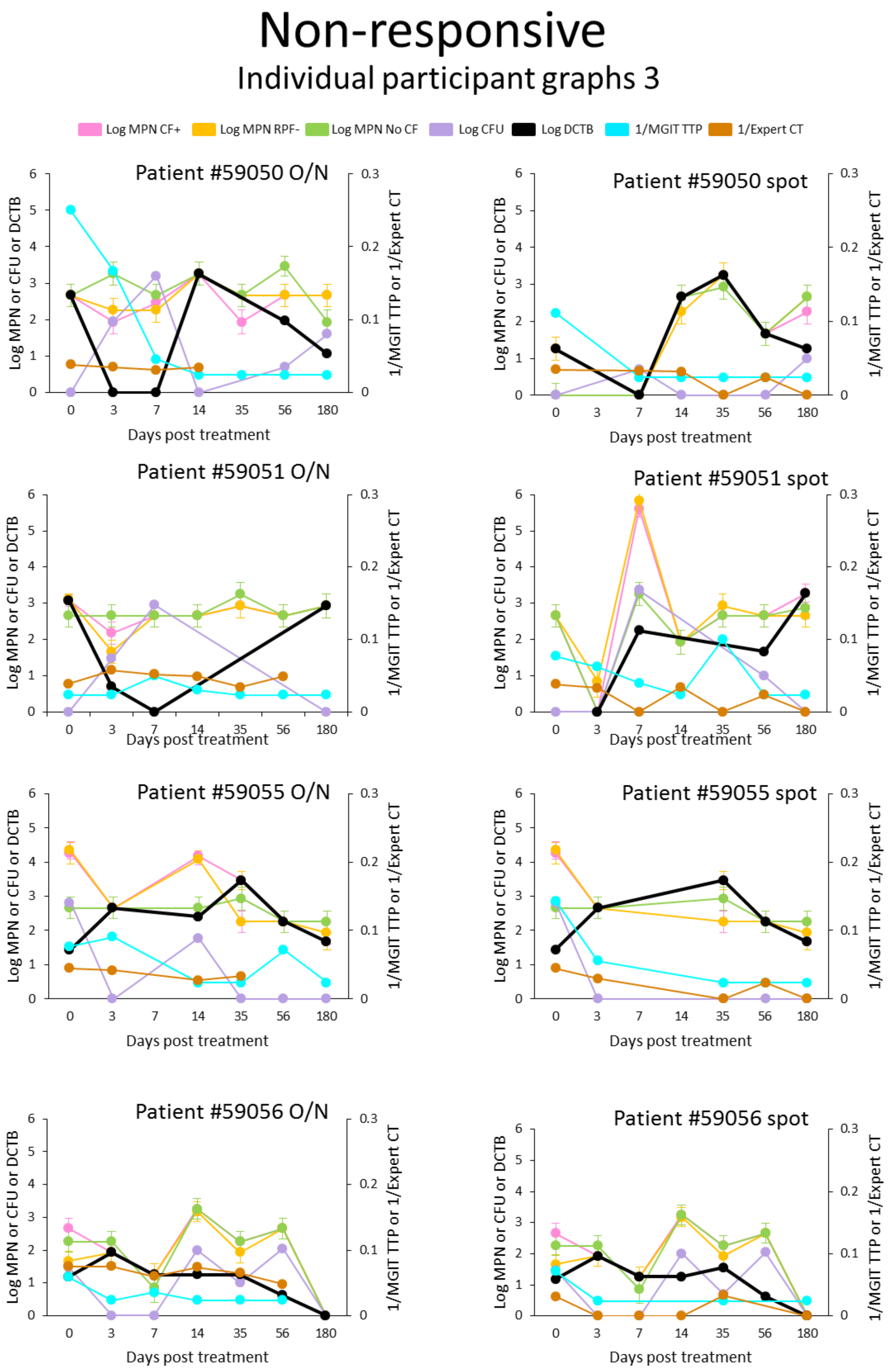
Non-responsive
Individual participant graphs 3
Log MPN CF+
Log MPN RPF-
Log MPN No CF
Log CFU
Log DCTB
1/MGIT TTP
1/Expert CT
Patient #59050 O/N
Patient #59050 spot
Patient #59051 O/N
Patient #59051 spot
Patient #59055 O/N
Patient #59055 spot
Patient #59056 O/N
Patient #59056 spot
Log MPN or CFU or DCTB
1/MGIT TTP or 1/Expert CT
Days post treatment
0
3
7
14
35
56
180

Non-responsive

Individual participant graphs 4

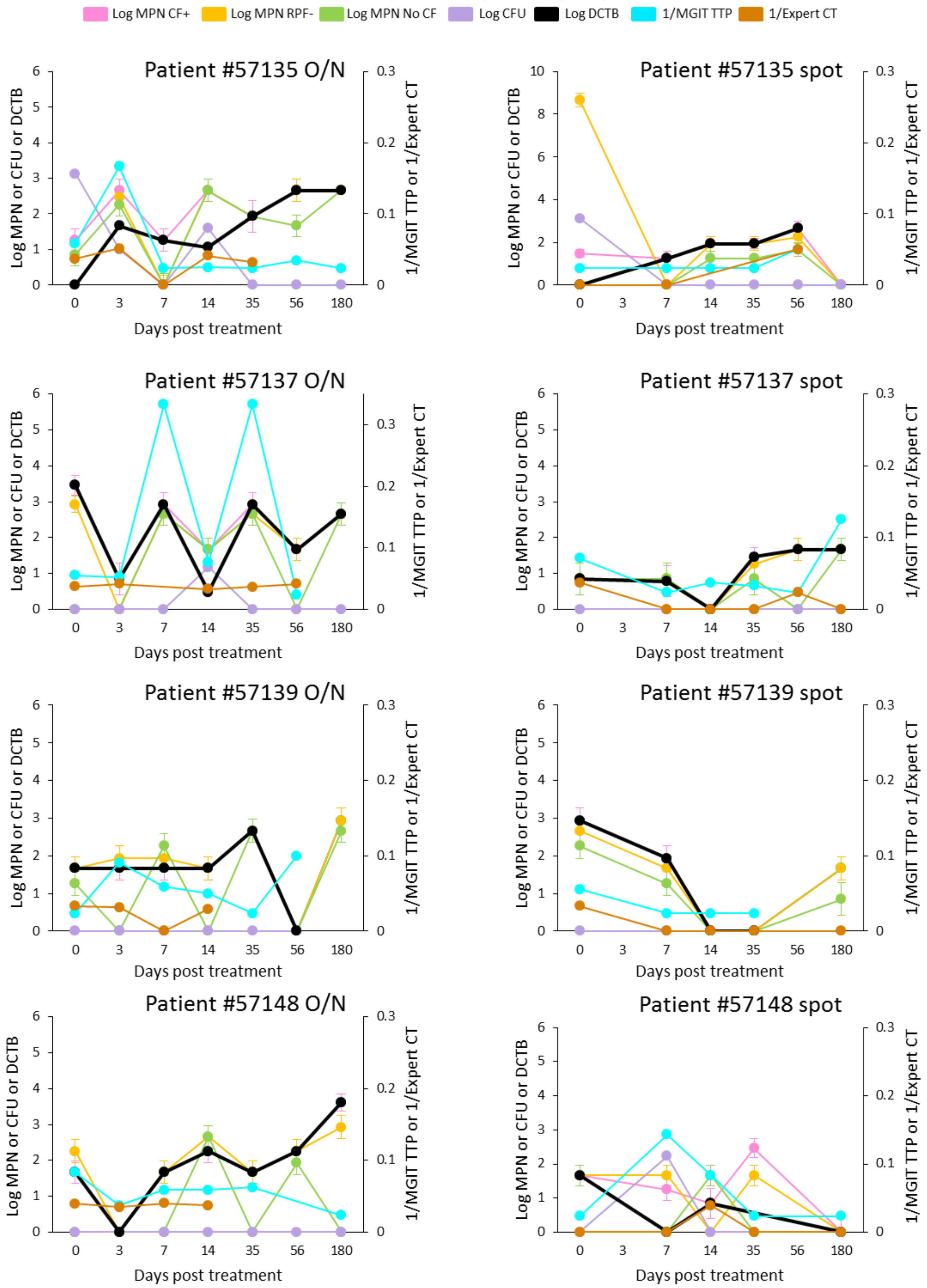

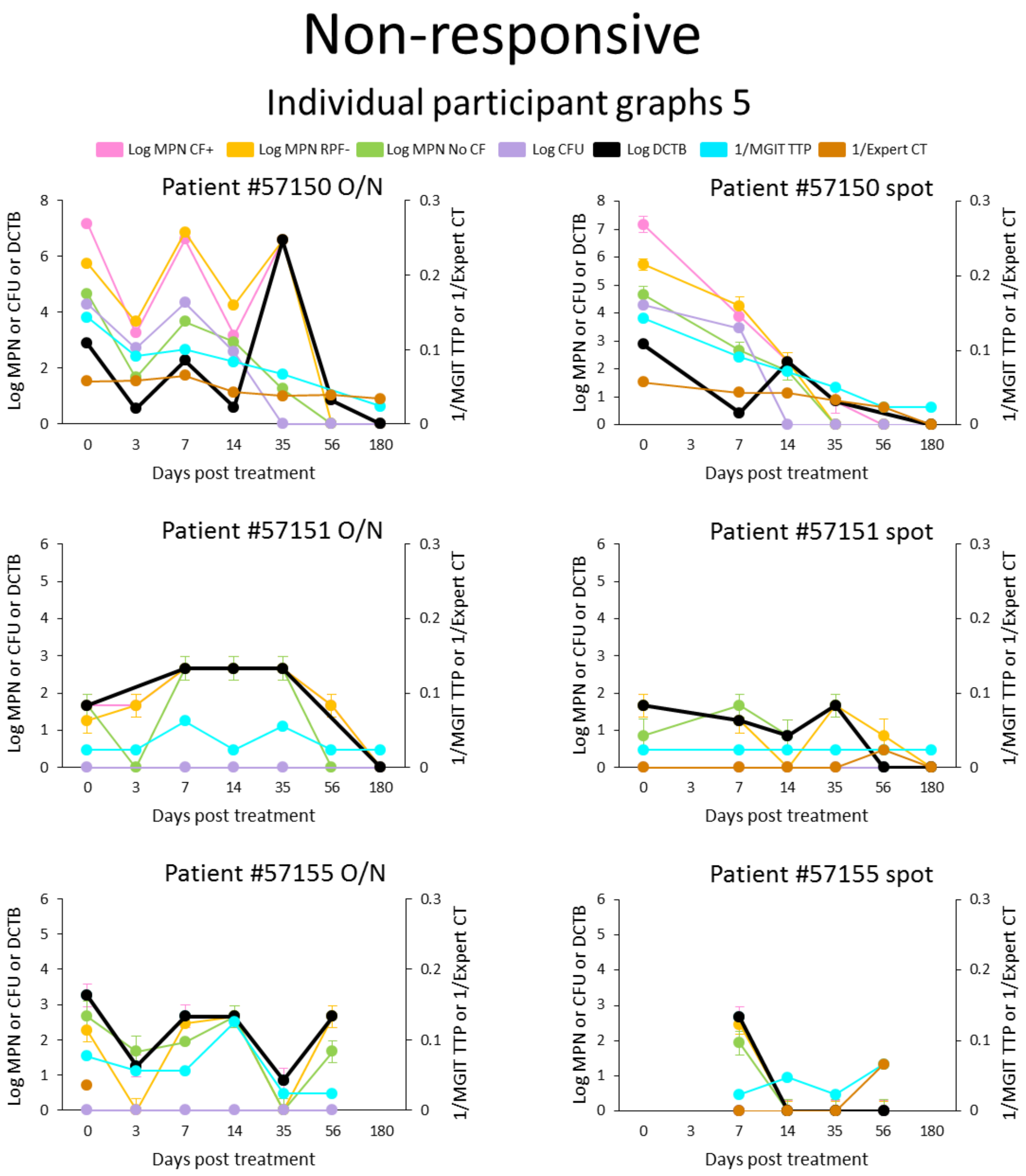
Non-responsive
Individual participant graphs 5
Log MPN CF+
Log MPN RPF-
Log MPN No CF
Log CFU
Log DCTB
1/MGIT TTP
1/Expert CT
Patient #57150 O/N
Patient #57150 spot
Patient #57151 O/N
Patient #57151 spot
Patient #57155 O/N
Patient #57155 spot
Log MPN or CFU or DCTB
1/MGIT TTP or 1/Expert CT
Days post treatment
0
3
7
14
35
56
180

References

1. Saito K, Warrier T, Somersan-Karakaya S, Kaminski L, Mi J, Jiang X, Park S, Shigyo K, Gold B, Roberts J, Weber E, Jacobs WR, Jr., Nathan CF. Rifamycin action on RNA polymerase in antibiotic-tolerant *Mycobacterium tuberculosis* results in differentially detectable populations. *Proc Natl Acad Sci U S A* 2017; 114: E4832-E4840.
2. Ioerger TR, Feng Y, Ganesula K, Chen X, Dobos KM, Fortune S, Jacobs WR, Mizrahi V, Parish T, Rubin E. Variation among genome sequences of H37Rv strains of Mycobacterium tuberculosis from multiple laboratories. *Journal of bacteriology* 2010; 192: 3645-3653.
3. Kana BD, Gordhan BG, Downing KJ, Sung N, Vostroktunova G, Machowski EE, Tsenova L, Young M, Kaprelyants A, Kaplan G, Mizrahi V. The resuscitation-promoting factors of *Mycobacterium tuberculosis* are required for virulence and resuscitation from dormancy but are collectively dispensable for growth in vitro. *Mol Microbiol* 2008; 67: 672-684.
4. Chengalroyen MD, Beukes GM, Gordhan BG, Streicher EM, Churchyard G, Hafner R, Warren R, Otwombe K, Martinson N, Kana BD. Detection and Quantification of Differentially Culturable Tubercle Bacteria in Sputum from Tuberculosis Patients. *American Journal of Respiratory And Critical Care Medicine* 2016.
5. Kamariza M, Shieh P, Ealand CS, Peters JS, Chu B, Rodriguez-Rivera FP, Babu Sait MR, Treuren WV, Martinson N, Kalscheuer R, Kana BD, Bertozzi CR. Rapid detection of *Mycobacterium tuberculosis* in sputum with a solvatochromic trehalose probe. *Sci Transl Med* 2018; 10.
